# The Triglyceride‐Glucose Index Combined With Obesity Indices and Lower Extremity Artery Disease in Type 2 Diabetes: A Sex‐Stratified Analysis

**DOI:** 10.1002/edm2.70278

**Published:** 2026-07-07

**Authors:** Rong Zhang, Guilin Liu, Qian Cui, Dan Fang, Zhenzhen Sun, Xiaodong Chen, Rendong Zheng, Zhenxiu Gao, Xiaodan Yuan

**Affiliations:** ^1^ School of Nursing, Nanjing University of Chinese Medicine Nanjing China; ^2^ Department of Endocrinology Affiliated Changzhou No. 2 People's Hospital of Nanjing Medical University Changzhou China; ^3^ Department of Endocrinology Affiliated Hospital of Nanjing University of Chinese Medicine Nanjing China; ^4^ Department of Cardiology Affiliated Hospital of Integrated Traditional Chinese and Western Medicine, Nanjing University of Chinese Medicine Nanjing China; ^5^ Department of Endocrinology Affiliated Hospital of Integrated Traditional Chinese and Western Medicine, Nanjing University of Chinese Medicine Nanjing China; ^6^ School of International Education, Nanjing Medical University Nanjing China; ^7^ Department of Public Health Affiliated Hospital of Integrated Traditional Chinese and Western Medicine, Nanjing University of Chinese Medicine Nanjing China

**Keywords:** lower extremity artery disease, obesity, triglyceride‐glucose index, type 2 diabetes mellitus

## Abstract

**Background:**

Lower extremity artery disease (LEAD) is highly prevalent among people with type 2 diabetes mellitus (T2DM) in China. This study aimed to investigate the associations of the triglyceride‐glucose (TyG) index and its combinations with obesity indicators with LEAD in patients with T2DM, and to evaluate their ability to discriminate LEAD.

**Methods:**

A total of 2424 individuals with T2DM were recruited from two tertiary hospitals in Jiangsu Province between June 2018 and July 2022. Restricted cubic spline (RCS) curves were used to evaluate the nonlinear associations of the TyG index and its combinations with obesity indicators with LEAD. Multivariate logistic regression analysis and receiver operating characteristic (ROC) curve analysis were performed to assess the association and discriminatory ability of these indices. Pairwise comparisons of AUCs were performed using the DeLong test, and Bonferroni correction was applied to adjust for multiple comparisons.

**Results:**

RCS analysis revealed three distinct association patterns: TyG‐BMI showed a positive linear association, the TyG index and TyG‐WHR exhibited U‐shaped associations, and TyG‐WC, TyG‐WHtR and TyG‐NC demonstrated J‐shaped associations with LEAD. In sex‐stratified analyses, most indices showed positive linear associations in males, whereas nonlinear patterns were observed for multiple indices in females. Among all indices, TyG‐BMI exhibited the highest discriminatory ability for identifying LEAD, albeit with a modest unadjusted AUC of 0.623. This discriminatory ability was more pronounced in males (AUC: 0.660) than in females (AUC: 0.539).

**Conclusion:**

Among the TyG‐derived indices, TyG‐BMI demonstrated the highest discriminatory ability for identifying LEAD in patients with T2DM, particularly in males; however, the discriminatory performance remained modest (AUC: 0.623). TyG‐BMI may serve as a convenient tool for rapid LEAD risk stratification using routine clinical data to identify individuals requiring further vascular assessment.

## Graphical abstract

TyG‐BMI demonstrated the highest discriminative ability for lower extremity artery disease (LEAD) in patients with T2DM (AUC = 0.623), outperforming the TyG index and other obesity‐combined indices. Its performance was notably better in males (AUC = 0.660). TyG‐BMI, calculated from routine clinical data, may serve as a convenient tool for rapid LEAD risk stratification, particularly in male T2DM patients.

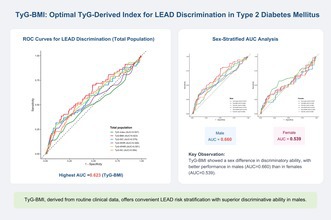

## 1 Introduction

Lower extremity artery disease (LEAD), the predominant manifestation of peripheral artery disease in patients with diabetes, represents a major macrovascular complication of type 2 diabetes mellitus (T2DM). A 2025 Global Burden of Disease Study reported 113.7 million prevalent cases of LEAD worldwide in 2021, with females accounting for 67.0% of all cases [1]. Notably, high fasting plasma glucose has become the leading modifiable risk factor for LEAD, contributing to 36.06% of LEAD‐related disability‐adjusted life years (DALYs) globally [2]. In China, the prevalence of LEAD among adults with T2DM reaches 21.2% [3], However, the diagnostic rate of LEAD in Chinese adults aged 50 years and older with T2DM is merely 11.8%, accompanied by a missed diagnosis rate of 55.7% [4]. Furthermore, public awareness of LEAD in this population ranges from only 16.6% to 33.9% [5]. Low diagnostic rates, high missed diagnosis rates and insufficient public awareness collectively result in delayed treatment and impede the implementation of preventive measures. Additionally, early clinical symptoms of LEAD are frequently atypical, causing many patients to miss the optimal window for diagnosis and intervention. LEAD is one of the leading causes of foot ulcers, subsequent disability and premature death in patients with T2DM, imposing a substantial burden on individuals, healthcare systems and society [6, 7, 8]. Therefore, identifying asymptomatic high‐risk individuals with LEAD using easily accessible indicators is of paramount importance for early intervention and improved clinical outcomes.

The ankle‐brachial index (ABI) is currently the gold standard for clinical screening of LEAD [9]. However, its application is not readily feasible in routine clinical practice within primary healthcare settings. The triglyceride‐glucose (TyG) index, a novel biomarker derived from fasting plasma glucose and triglycerides, has been established as a simple, reliable and cost‐effective surrogate for insulin resistance (IR) [10]. Its ease of measurement, affordability and wide applicability make it a valuable tool in clinical practice.

Previous studies have demonstrated that both obesity and the TyG index are independently associated with an increased risk of LEAD [11, 12]. Nevertheless, the combined association of the TyG index and obesity indices with LEAD in patients with T2DM has not been extensively investigated. Therefore, this study aims to investigate the independent and combined associations of the TyG index and obesity indices with LEAD in patients with T2DM, and to evaluate the discriminatory ability of these indicators to identify asymptomatic individuals with LEAD.

## 2 Methods

### 2.1 Study Population

This cross‐sectional study involved 2424 participants with T2DM from the endocrinology departments of two tertiary hospitals in Jiangsu Province, enrolled between June 2018 and July 2022. The inclusion criteria were a diagnosis of T2DM according to the 1999 World Health Organization (WHO) criteria and age ≥ 18 years. The exclusion criteria were: (1) a history of stroke, coronary heart disease or other macrovascular diseases; (2) malignant tumours, autoimmune diseases, haematological disorders or pregnancy; (3) heart failure, severe hepatic or renal disease or a history of thrombolytic therapy or lower extremity revascularization. Abnormal ABI was defined according to the screening criteria for LEAD in the diabetic population specified in the “Guideline for the Prevention and Treatment of Type 2 Diabetes Mellitus in China (2024 edition)” [13]. LEAD was diagnosed when the ABI of either lower extremity was ≤ 0.9 or ≥ 1.3, with evidence of vascular abnormalities on imaging. Of the 2424 participants, 2253 did not have LEAD and 171 were diagnosed with the condition. This study was approved by the Ethics Committee of the Affiliated Hospital of Nanjing University of Chinese Medicine (approval No. 2021‐LWKY‐020), and all participants provided written informed consent before enrollment.

### 2.2 Demographic and Clinical Characteristics

Standardized questionnaires were used to collect demographic information, including age, sex, educational attainment and health‐related behaviours such as smoking status and alcohol consumption. History of hypertension and hyperlipidemia, as well as use of antihypertensive and lipid‐lowering medications, were documented. Physical activity and sedentary behaviour were assessed using the International Physical Activity Questionnaire‐Short Form (IPAQ‐SF) [14]. Weekly physical activity (PA) was calculated as metabolic equivalent of task (MET) × daily activity duration (min/d) × weekly frequency of activity (d/w). MET values for walking, moderate PA and vigorous PA were assigned as 3.3, 4.0 and 8.0, respectively. Daily sedentary time was calculated as (weekday sitting time × 5 + weekend sitting time × 2)/7, excluding time spent sleeping or napping.

### 2.3 Anthropometric and Laboratory Data

Height and weight were measured with participants barefoot, wearing light clothing and without headgear, using a medical scale (Omron HNH‐318). Waist circumference (WC) was measured at the end of expiration with a non‐elastic tape measure, positioned at the midpoint between the lower rib margin and the upper iliac crest, to an accuracy of 0.1 cm. Neck circumference (NC) was measured at the level of the laryngeal prominence with a non‐elastic tape measure applied snugly against the skin, while participants stood naturally and breathed calmly. Body mass index (BMI) was calculated as weight (kilograms) divided by the square of height (metres). Systolic blood pressure (SBP) and diastolic blood pressure (DBP) were measured in the right arm using an electronic blood pressure monitor (Omron HBP‐1100 U). Three consecutive readings were obtained, and the average value was used for analysis.

Venous blood samples were collected after an overnight fast of more than 8 h. Fasting plasma glucose (FPG), total cholesterol (TC), triglycerides (TG), low‐density lipoprotein cholesterol (LDL‐c) and high‐density lipoprotein cholesterol (HDL‐c) were measured using an automatic biochemical analyser (Roche Cobas C702). Glycated haemoglobin (HbA1c) was determined by high‐performance liquid chromatography (HPLC) using the Bio‐Rad D‐10 system. Serum creatinine was measured using the same automatic biochemical analyser to calculate estimated glomerular filtration rate (eGFR) via the Chronic Kidney Disease Epidemiology Collaboration (CKD‐EPI) equation [15]. Urinary microalbumin and urinary creatinine were assayed to derive the urine albumin‐to‐creatinine ratio (UACR), expressed in mg/g as follows: UACR (mg/g) = urinary microalbumin (mg/L)/urinary creatinine (g/L).

Blood and urine samples were collected in vacuum tubes, centrifuged and stored at −80°C within 2 h of collection.

### 2.4 ABI Measurement

The Omron BP‐203RPE III device was used for arteriosclerosis assessment. Participants rested in the supine position for 10–15 min before measurement. Cuffs were placed on both upper arms over the brachial arteries and 2–3 cm above the medial malleolus of both ankles. After entering the relevant participant data, the device automatically calculated the ABI. Bilateral ABI values were recorded for analysis.

### 2.5 Definition of the TyG Index and Its Combinations With Obesity Indices

1. TyG index = ln [(triglycerides (mg/dL) × fasting plasma glucose (mg/dL))/2];
2. BMI = body mass (kg)/height^2^ (m^2^);
3. WHtR = waist circumference (WC) / height; WHR = waist circumference (WC) / hip circumference (HC);
4. TyG‐BMI = TyG index × BMI; TyG‐WHtR = TyG index × WHtR; TyG‐WHR = TyG index × WHR; TyG‐WC = TyG index × WC; TyG‐NC = TyG index × NC.

### 2.6 Statistical Analysis

Baseline characteristics of participants were presented as median (interquartile range) for continuous variables and number (percentage) for categorical variables. Differences between male and female participants were examined using the Mann–Whitney *U* test for continuous variables and the chi‐square test for categorical variables.

Multivariate logistic regression models were constructed to explore the independent association between the TyG index and its combinations with obesity indices and LEAD in patients with T2DM. Odds ratios (ORs) and corresponding 95% confidence intervals (CIs) were calculated. Variance inflation factor (VIF) analysis was performed to assess multicollinearity among variables, with a VIF < 5 indicating no significant multicollinearity. Sequential models were constructed to assess the independent associations and potential effect modification of sex. For the total population, Model 1 was adjusted for sex, age, educational attainment, duration of diabetes, smoking status, alcohol consumption, hypertension, hyperlipidemia, use of lipid‐lowering medication, HbA1c, HDL‐c, LDL‐c, DBP, SBP, UACR, eGFR, physical activity and sedentary behaviour. To further evaluate whether sex modifies the strength of these associations, Model 2 included an additional “index × sex” interaction term, and the Wald test was used to calculate the *p*‐value for the interaction term. In addition, we performed sex‐stratified analyses to examine sex‐specific associations, using the same covariate set as Model 1 except for sex, which was not adjusted for as it served as the stratification variable.

Restricted cubic spline (RCS) curves were used to evaluate the nonlinear association of each TyG‐derived index with LEAD, with knots placed at the 5th, 50th and 95th percentiles of the distribution of the independent variable. For indices confirmed to have a nonlinear association via RCS analysis, optimal cut‐off values were determined based on the intersection points of the RCS curves and the OR = 1 reference line and the study population was stratified accordingly. Within each stratum, the index was entered as a continuous variable into the multivariate logistic regression model to analyse its independent association with LEAD in different intervals.

Receiver operating characteristic (ROC) curves were plotted using the pROC package in R software to evaluate the discriminatory ability of each index for LEAD in patients with T2DM. The area under the curve (AUC) and corresponding 95% CIs were calculated. The automatic direction detection function of the pROC package was enabled to correct for AUC values below 0.5 (i.e., when index values were lower in the case group than in the control group in some subgroups). Pairwise comparisons of AUCs were performed using the DeLong test, and Bonferroni correction was applied to adjust for multiple comparisons.

All statistical analyses were performed using R software (version 4.4.0). A two‐tailed *p*‐value < 0.05 was considered statistically significant.

## 3 Results

### 3.1 Baseline Characteristics of the Study Population

A total of 2424 participants with T2DM were included in this study, among whom 171 (7.1%) were diagnosed with LEAD. The median age of the overall population was 53 years.

Male participants had significantly higher educational attainment (high school or above), current smoking rate, current alcohol consumption rate, physical activity levels, DBP, FPG, TG, BMI, WC, HC, NC, WHR and all TyG‐related indices (TyG index, TyG‐BMI, TyG‐WC, TyG‐WHR, TyG‐WHtR, TyG‐NC) compared with female participants (all *p* < 0.05).

In contrast, female participants were older, had a longer duration of diabetes, a higher proportion of hypertension, higher levels of TC, HDL‐c and UACR, and a higher proportion of antihypertensive medication use than male participants (all *p* < 0.005).

No significant differences were observed between the two groups in proportion of hyperlipidemia, proportion of LEAD, use of lipid‐lowering medication, sedentary time, SBP, HbA1c, LDL‐c, eGFR or WHtR (all *p* > 0.05) (Table 1).

**TABLE 1:** Baseline characteristics of participants.

Characteristic	Total (*N* = 2424)	Male (*N* = 1593)	Female (*N* = 831)	*p*
Age, years	53 (44–58)	51 (43–57)	55 (48–59)	< 0.001
educational attainment (≥ high school)	1092 (45.0)	825 (51.8)	267 (32.1)	< 0.001
Duration of diabetes, months	68 (9–126)	62 (7–124)	83 (12–128)	< 0.001
Current smoker	1239 (51.1)	1227 (77.0)	12 (1.4)	< 0.001
Current alcohol consumption	1281 (52.8)	1212 (76.1)	69 (8.3)	< 0.001
Hypertension	1002 (41.3)	618 (38.8)	384 (46.2)	< 0.001
Hyperlipidemia	672 (27.7)	441 (27.7)	231 (27.8)	0.952
Antihypertensive medication	900 (37.1)	555 (34.8)	345 (41.5)	0.001
Lipid‐lowering medication	252 (10.4)	159 (10.0)	93 (11.2)	0.354
LEAD	171 (7.1)	123 (7.7)	48 (5.8)	0.076
Physical activity (MET), min/w	1386 (693–2772)	1397 (770–2663)	1359 (734–2081)	< 0.001
Sedentary time, h/d	4.71 (4.00–6.00)	4.73 (3.95–5.94)	4.71 (3.92–5.88)	0.061
DBP, mmHg	76 (69–82)	76 (70–83)	74 (67–81)	< 0.001
SBP, mmHg	125 (115–136)	125 (115–136)	125 (116–136)	0.805
FPG, mg/dL	142.74 (111.24–188.64)	143.82 (114.3–187.74)	140.22 (106.56–190.80)	0.043
HbA1c, %	9.0 (7.5–10.8)	8.9 (7.6–10.8)	9.0 (7.3–10.8)	0.309
TG, mg/dL	153.11 (106.20–238.95)	158.42 (108.86–254.00)	147.80 (100.89–210.63)	< 0.001
TC, mg/dL	175.24 (151.31–205.35)	173.31 (149.00–200.72)	180.26 (155.17–212.30)	0.001
LDL‐c, mg/dL	105.00 (84.53–127.38)	105.00 (84.92–126.61)	105.00 (80.67–127.38)	> 0.999
HDL‐c, mg/dL	38.98 (33.97–46.32)	37.83 (32.42–44.39)	42.46 (36.28–50.18)	< 0.001
eGFR, mL/(min·1.73m^2^)	103.79 (96.52–112.75)	103.78 (96.53–113.41)	104.22 (96.49–111.40)	0.385
UACR, mg/g	11.31 (6.60–31.29)	10.94 (6.06–28.57)	12.36 (7.57–38.91)	< 0.001
BMI, kg/m^2^	25.40 (23.50–27.73)	25.58 (23.79–27.82)	24.97 (22.98–27.45)	< 0.001
WC, cm	90.0 (85.0–96.0)	92.0 (87.0–98.0)	86.0 (81.0–92.5)	< 0.001
HC, cm	96.0 (92.1–101.0)	97.0 (93.0–101.0)	94.0 (91.0–100)	< 0.001
NC, cm	38.5 (36.0–40.8)	39.8 (38.0–41.5)	35.0 (33.5–37.0)	< 0.001
WHR	0.94 (0.90–0.98)	0.95 (0.91–0.99)	0.91 (0.87–0.96)	< 0.001
WHtR	0.54 (0.512–0.58)	0.55 (0.51–0.58)	0.54 (0.51–0.58)	0.590
TyG index	9.35 (8.85–9.86)	9.40 (8.85–9.94)	9.28 (8.84–9.77)	0.001
TyG‐BMI	237.33 (214.86–267.40)	239.55 (217.03–272.73)	230.86 (210.47–263.25)	< 0.001
TyG‐WC	844.00 (765.70–936.35)	868.97 (785.58–958.77)	797.76 (730.96–889.85)	< 0.001
TyG‐NC	359.07 (324.15–392.19)	373.47 (343.08–407.42)	325.71 (301.18–358.08)	< 0.001
TyG‐WHR	8.76 (8.04–9.53)	8.94 (8.20–9.63)	8.43 (7.79–9.12)	< 0.001
TyG‐WHtR	5.09 (4.62–5.64)	5.16 (4.66–5.67)	5.04 (4.57–5.61)	0.005

*Note:* Values are presented as median (interquartile range) or number (%).

Abbreviations: BMI, body mass index; DBP, diastolic blood pressure; FPG, fasting plasma glucose; HbA1c, glycosylated haemoglobin; HC, hip circumference; HDL‐c, high‐density lipoprotein cholesterol; LDL‐c, low‐density lipoprotein cholesterol; LEAD, lower extremity artery disease; MET, metabolic equivalent; NC, neck circumference; SBP, systolic blood pressure; TG, triglycerides; TyG‐BMI, triglyceride‐glucose index‐body mass index; TyG index, triglyceride‐glucose index; TyG‐NC, triglyceride‐glucose index‐neck circumference; TyG‐WC, triglyceride‐glucose index‐waist circumference; TyG‐WHR, triglyceride‐glucose index‐waist‐to‐height ratio; TyG‐WHtR, triglyceride‐glucose index‐waist‐to‐height ratio; WC, waist circumference; WHR, waist‐to‐hip ratio; WHtR, waist‐to‐height ratio.

### 3.2 Nonlinear Association of TyG Index and Its Combinations With Obesity Indices With LEAD in Patients With T2DM

**FIGURE 1:**
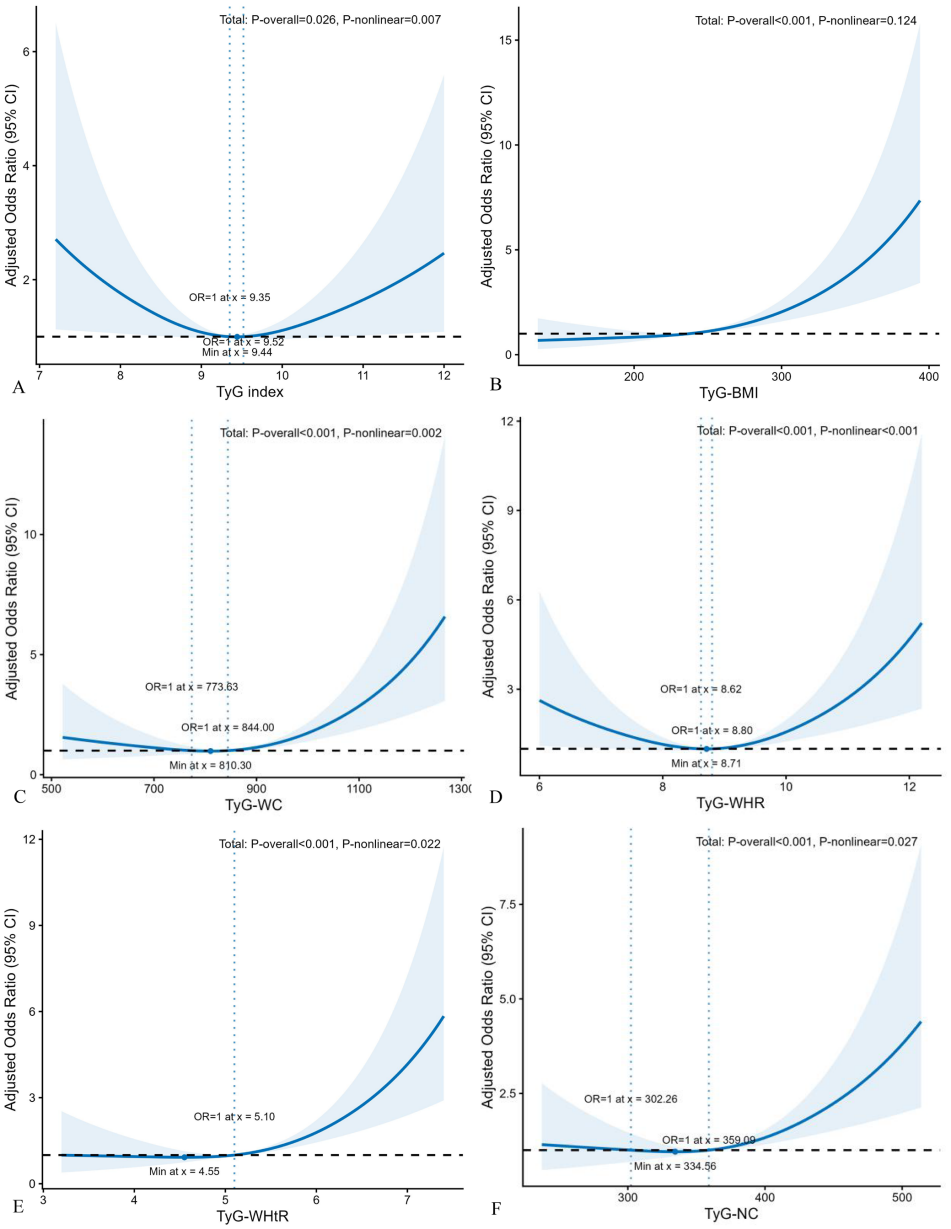
Restricted cubic spline curves for association of TyG index and its combinations with obesity indices with LEAD (total population) *Note*: Adjusted for sex, age, educational attainment, duration of diabetes, smoking status, alcohol consumption, hypertension, hyperlipidemia, lipid‐lowering medication, HbA1c, HDL‐c, LDL‐c, DBP, SBP, UACR, eGFR, physical activity and sedentary time.

In the total population, significant nonlinear associations between TyG index, TyG‐WC, TyG‐WHR, TyG‐WHtR and TyG‐NC with LEAD were observed (all *p* for nonlinear < 0.05), while TyG‐BMI showed a linear association (*p* for nonlinear > 0.05) (Figure 1A–1F). For indices with nonlinear associations, the OR = 1 reference line intersected the RCS curves at two cut‐off points, with the lowest OR values observed at the midpoints: TyG index at 9.44 (cut‐offs: 9.35 and 9.52), TyG‐WC at 810.30 (cut‐offs: 773.63 and 844.00), TyG‐WHR at 8.71 (cut‐offs: 8.62 and 8.80), TyG‐WHtR at 4.55 (upper cut‐off: 5.10) and TyG‐NC at 334.56 (cut‐offs: 302.26 and 359.09).

In sex‐stratified analyses, only TyG‐WHR exhibited a significant nonlinear association with LEAD in males (*p* for nonlinear = 0.023), while all other indices showed linear associations (all *p* for nonlinear > 0.05). In contrast, significant nonlinear associations were found for TyG index, TyG‐BMI, TyG‐WC, TyG‐WHR and TyG‐NC in females (all *p* for nonlinear < 0.05), whereas only TyG‐WHtR demonstrated a linear relationship (*p* for nonlinear > 0.05) (Figure S1).

### 3.3 Association of TyG Index and Its Combinations With Obesity Indices With LEAD and Sex Interaction Analysis in Patients With T2DM

In the total population, multivariate logistic regression analysis was performed with adjustment for all covariates in Model 1. TyG‐BMI showed a significant positive linear association with LEAD (OR = 1.010, 95% CI: 1.007–1.014, *p* < 0.001). For indices with nonlinear associations, significantly increased odds of LEAD were observed in participants with TyG ≥ 9.52 (OR = 1.672, 95% CI: 1.056–2.595, *p* = 0.024), TyG‐WC ≥ 844.00 (OR = 1.004, 95% CI: 1.002–1.007, *p* < 0.001), TyG‐WHR ≥ 8.80 (OR = 1.796, 95% CI: 1.312–2.449, *p* < 0.001), TyG‐WHtR > 5.10 (OR = 2.524, 95% CI: 1.683–3.794, *p* < 0.001), TyG‐NC 302.26–359.09 (OR = 1.023, 95% CI: 1.000–1.047, *p* = 0.049) and TyG‐NC ≥ 359.09 (OR = 1.007, 95% CI: 1.002–1.013, *p* = 0.011). TyG‐WHR ≤ 8.62 was associated with significantly decreased odds of LEAD (OR = 0.563, 95% CI: 0.346–0.921, *p* = 0.021). Interaction analysis revealed significant effect modification of sex on the associations of TyG index (*p* for interaction = 0.019) and TyG‐WHR (*p* for interaction = 0.024) with LEAD (Table 2).

**TABLE 2:** Association of TyG index and its combinations with obesity indices with LEAD and sex interaction in patients with T2DM.

Variable	*N* (Events)	Unadjusted		Model 1		Model 2		
		OR (95% CI)	*p*	OR (95% CI)	*p*	OR (95% CI)	*p*	*p* for interaction
TyG ≤ 9.35	1212 (84)	0.628 (0.408, 0.986)	0.039	0.949 (0.549, 1.662)	0.852	1.506 (0.762, 3.097)	0.251	0.019
TyG ≥ 9.52	978 (78)	1.371 (0.924, 1.973)	0.101	1.672 (1.056, 2.595)	0.024	1.701 (1.029, 2.744)	0.033	0.866
TyG 9.35–9.52	234 (9)	—	—	—	—	—	—	—
TyG‐BMI	2424 (171)	1.009 (1.006, 1.012)	< 0.001	1.010 (1.007, 1.014)	< 0.001	1.012 (1.007, 1.016)	< 0.001	0.159
TyG‐WC ≤ 773.63	669 (39)	0.996 (0.991, 1.001)	0.083	0.998 (0.991, 1.005)	0.530	0.997 (0.987, 1.006)	0.467	0.707
TyG‐WC ≥ 844.00	1212 (102)	1.004 (1.002, 1.006)	< 0.001	1.004 (1.002, 1.007)	< 0.001	1.004 (1.002, 1.006)	0.001	0.611
TyG‐WC 773.63–844.00	543 (30)	0.997 (0.980, 1.014)	0.729	0.990 (0.969, 1.011)	0.342	0.990 (0.967, 1.012)	0.356	0.922
TyG‐WHR ≤ 8.62	1128 (69)	0.630 (0.439, 0.922)	0.014	0.563 (0.346, 0.921)	0.021	0.794 (0.445, 1.471)	0.447	0.024
TyG‐WHR ≥ 8.80	1128 (87)	1.673 (1.278, 2.167)	< 0.001	1.796 (1.312, 2.449)	< 0.001	1.713 (1.214, 2.402)	0.002	0.487
TyG‐WHR 8.62–8.80	168 (15)	—	—	—	—	—	—	—
TyG‐WHtR ≤ 5.10	1272 (72)	0.981 (0.567, 1.768)	0.946	1.313 (0.655, 2.714)	0.452	1.668 (0.724, 4.068)	0.244	0.304
TyG‐WHtR > 5.10	1152 (99)	2.041 (1.454, 2.840)	< 0.001	2.524 (1.683, 3.794)	< 0.001	2.636 (1.679, 4.138)	< 0.001	0.666
TyG‐NC ≤ 302.26	294 (24)	0.998 (0.978, 1.020)	0.829	0.929 (0.592, 1.104)	0.567	—	—	—
TyG‐NC ≥ 359.09	1215 (105)	1.008 (1.004, 1.013)	< 0.001	1.007 (1.002, 1.013)	0.011	1.007 (1.001, 1.013)	0.027	0.697
TyG‐NC 302.26–359.09	915 (42)	1.018 (0.998, 1.039)	0.078	1.023 (1.000, 1.047)	0.049	1.040 (1.010, 1.074)	0.012	0.087

*Note:* Model 1 was adjusted for sex, age, educational attainment, duration of diabetes, hypertension, hyperlipidemia, lipid‐lowering medication, smoking status, alcohol consumption, HbA1c, HDL‐c, LDL‐c, DBP, SBP, UACR, eGFR, physical activity and sedentary behaviour. Model 2 further added the “index × sex” interaction term based on Model 1. “—” indicates that data are not presented because the model failed to provide stable estimates due to insufficient sample size or too few outcome events in that stratum. *p‐*values for interaction were derived from the Wald test of the product term (variable × sex) in Model 2.

*Abbreviation*s: CI, confidence interval; OR, odds ratio.

In sex‐stratified analyses (Table S1), in males, TyG‐BMI (OR = 1.012, 95% CI: 1.007–1.016, *p* < 0.001), TyG‐WC (OR = 1.003, 95% CI: 1.001–1.005, *p* < 0.001), TyG‐WHR ≥ 8.90 (OR = 1.912, 95% CI: 1.308–2.793, *p* < 0.001), TyG‐WHtR (OR = 1.765, 95% CI: 1.353–2.307, *p* < 0.001) and TyG‐NC (OR = 1.008, 95% CI: 1.003–1.012, *p* < 0.001) showed significant positive associations with LEAD. No significant association was observed for TyG index. In females, only TyG‐BMI ≥ 232.47 (OR = 1.022, 95% CI: 1.009–1.036, *p* = 0.001) and TyG‐WHtR (OR = 1.616, 95% CI: 1.032–2.520, *p* = 0.034) were significantly associated with increased odds of LEAD. No significant associations were found for TyG index, TyG‐WC, TyG‐WHR or TyG‐NC.

### 3.4 Discriminatory Ability of TyG Index and Its Combinations With Obesity Indices for LEAD in Patients With T2DM

**FIGURE 2:**
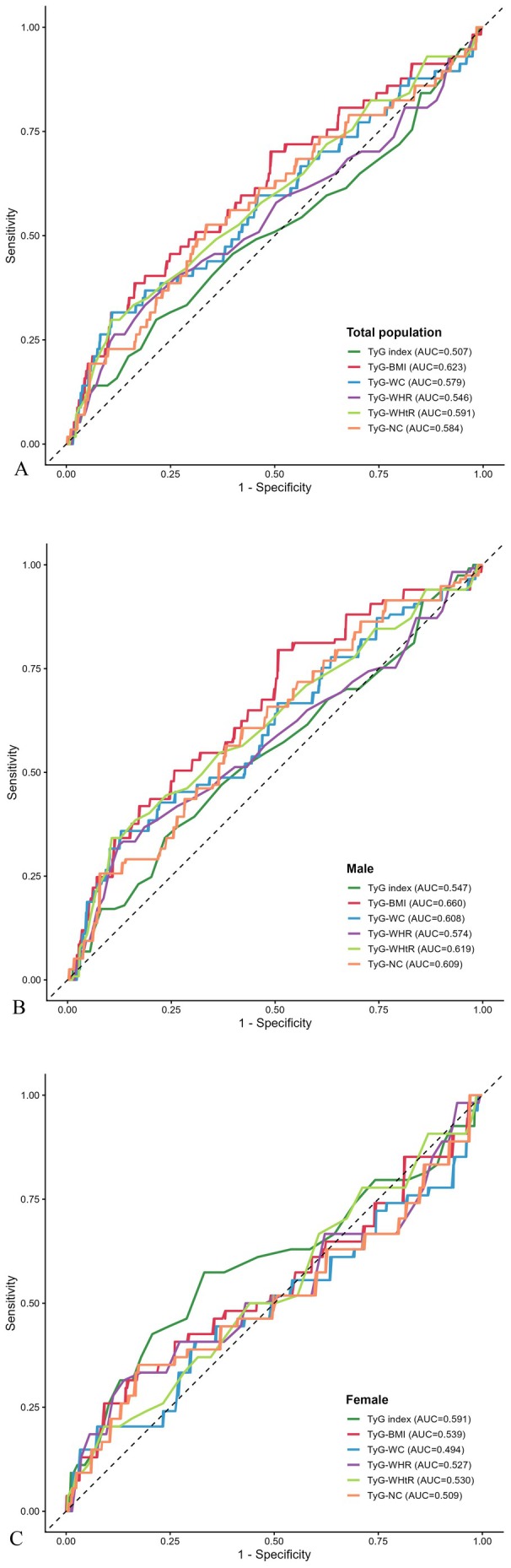
Receiver operating characteristic curves showing discriminatory ability of TyG index and its combinations with obesity indices for LEAD (A: Total population; B: Male; C: Female).

In the total population, the unadjusted model showed that TyG‐BMI had the highest discriminatory ability with an AUC of 0.623, followed by TyG‐WHtR (0.591), TyG‐NC (0.584), TyG‐WC (0.579), TyG‐WHR (0.546) and TyG index (0.507). Pairwise comparisons revealed that the AUC of TyG‐BMI was significantly higher than those of TyG index, TyG‐WC and TyG‐WHR (all Bonferroni *p* < 0.05) (Figure 2, Table 3 and Table S2).

**TABLE 3:** Discriminatory ability of TyG index and its combinations with obesity indices for LEAD in patients with T2DM.

Index	Total (*N* = 2424)			Male (*N* = 1593)			Female (*N* = 831)		
	AUC	95% CI	*p*	AUC	95% CI	*p*	AUC	95% CI	*p*
TyG index	0.507	0.457–0.556	0.394	0.547	0.490–0.604	0.053	0.591	0.499–0.683	0.027
*TyG index adj*.	*0.670*	*0.627–0.712*	*< 0.001*	*0.669*	*0.617–0.721*	*< 0.001*	*0.756*	*0.683–0.829*	*< 0.001*
TyG‐BMI	0.623	0.576–0.670	< 0.001	0.660	0.607–0.712	< 0.001	0.539	0.447–0.632	0.202
*TyG‐BMI adj*.	*0.699*	*0.658–0.739*	*< 0.001*	*0.706*	*0.658–0.755*	*< 0.001*	*0.768*	*0.693–0.843*	*< 0.001*
TyG‐WC	0.579	0.530–0.628	< 0.001	0.608	0.551–0.665	< 0.001	0.494	0.401–0.586	0.554
*TyG‐WC adj*.	*0.682*	*0.640–0.724*	*< 0.001*	*0.678*	*0.626–0.729*	*< 0.001*	*0.763*	*0.690–0.837*	*< 0.001*
TyG‐WHR	0.546	0.495–0.597	0.039	0.574	0.514–0.634	0.008	0.527	0.433–0.621	0.287
*TyG‐WHR adj*.	*0.672*	*0.629–0.715*	*< 0.001*	*0.668*	*0.616–0.721*	*< 0.001*	*0.759*	*0.684–0.834*	*< 0.001*
TyG‐WHtR	0.591	0.543–0.639	< 0.001	0.619	0.561–0.676	< 0.001	0.530	0.444–0.615	0.248
*TyG‐WHtR adj*.	*0.689*	*0.647–0.731*	*< 0.001*	*0.684*	*0.632–0.735*	*< 0.001*	*0.772*	*0.700–0.843*	*< 0.001*
TyG‐NC	0.584	0.536–0.632	< 0.001	0.609	0.556–0.663	< 0.001	0.509	0.415–0.603	0.425
*TyG‐NC adj*.	*0.686*	*0.646–0.727*	*< 0.001*	*0.682*	*0.631–0.733*	*< 0.001*	*0.761*	*0.687–0.834*	*< 0.001*

*Note:* adj. indicates adjusted model. Model was adjusted for sex, age, educational attainment, duration of diabetes, smoking status, alcohol consumption, hypertension, hyperlipidemia, lipid‐lowering medication, HbA1c, HDL‐c, LDL‐c, DBP, SBP, UACR, eGFR, physical activity and sedentary time. Sex‐stratified models were not adjusted for sex.

*Abbreviation*s: AUC, area under the ROC curve; ROC, receiver operating characteristic.

In sex‐stratified analyses, the unadjusted AUC of TyG‐BMI in males was 0.660, which was significantly superior to all five other indices (all Bonferroni *p* < 0.05). In females, only the unadjusted AUC of the TyG index was statistically significant (*p* = 0.027), and no significant differences were observed between the other indices (Figure 2, Table 3 and Table S2).

After adjustment for all covariates, the AUCs of all indices increased significantly compared with the unadjusted models (all *p* < 0.05). As presented in Figure S2, the adjusted AUC was highest for TyG‐BMI in both the total population (0.699) and the male population (0.706). Although the differences in adjusted AUCs between indices were smaller than those in the unadjusted models, the adjusted AUC of TyG‐BMI remained significantly higher than those of TyG‐WC, TyG‐WHR, TyG‐WHtR and TyG‐NC in males (all Bonferroni *p* < 0.05). In females, the highest adjusted AUC was observed for TyG‐WHtR (0.772), followed by TyG‐BMI (0.768) and TyG‐WC (0.763). No significant differences in adjusted AUCs were found in any Bonferroni‐corrected pairwise comparisons between indices in either the total population or females (Table 3 and Table S3).

## 4 Discussion

Obesity, IR and the development of atherosclerosis are closely interrelated. The TyG index, a reliable surrogate for IR, has been widely used in clinical research. However, the combined associations of the TyG index with various obesity indices and LEAD in patients with T2DM remain to be fully explored. In this study of adults with T2DM, we systematically evaluated the association patterns and discriminatory ability of the TyG index and its combinations with five obesity indices for LEAD, and further examined sex‐specific differences in these associations.

In the present study, RCS analysis revealed three distinct patterns of association between TyG‐derived indices and LEAD. TyG‐BMI showed a linear positive association. Multivariate logistic regression indicated that each 1‐unit increase in TyG‐BMI was associated with a 1.0% increase in the odds of LEAD. This finding is consistent with Zheng et al. [16], who similarly observed a linear association between TyG‐BMI and coronary heart disease risk in individuals with prediabetes and diabetes. LEAD and coronary artery disease share common pathological mechanisms, including endothelial dysfunction and oxidative stress. As a composite index integrating IR and overall obesity, TyG‐BMI captures both the severity of insulin resistance and the burden of adiposity, thereby providing a more comprehensive assessment of current metabolic vascular damage and LEAD risk.

TyG index and TyG‐WHR exhibited U‐shaped associations with LEAD. The RCS curve for the TyG index intersected the OR = 1 reference line at two cut‐off values (9.35 and 9.52), with the lowest point at 9.44. A TyG index ≥ 9.52 was associated with a 67.2% increase in the odds of LEAD, whereas no significant association was observed below this threshold. Zhao et al. [17] reported no association between the TyG index (mean 8.75) and lower extremity atherosclerosis in a community‐dwelling elderly population in Shanghai; this mean value falls below our upper cut‐off of 9.52, corroborating our finding that the risk does not increase at lower TyG index levels. Ning et al. [18] reported that each 1‐unit increase in the TyG index was associated with a 3.92‐fold increase in the odds of LEAD in patients with diabetes, with a mean TyG index of 9.45, which approaches our inflection point of 9.44, further supporting the notion that the risk increases substantially beyond a specific threshold. For TyG‐WHR, the RCS curve intersected the reference line at two cut‐off values (8.62 and 8.80), with an inflection point at 8.71. A TyG‐WHR ≤ 8.62 was associated with a 43.7% decrease in the odds of LEAD, whereas a TyG‐WHR ≥ 8.80 was associated with an approximately 1.80‐fold increase. This U‐shaped pattern can be explained by differences in fat distribution. WHR is the ratio of WC to HC: a larger HC relative to WC yields a lower WHR, reflecting predominant gluteofemoral subcutaneous fat, which exerts metabolically protective effects and is associated with lower risks of cardiovascular disease and diabetes [19]; conversely, a larger WC relative to HC yields a higher WHR, reflecting visceral fat accumulation, which releases substantial amounts of free fatty acids into the circulation, induces dyslipidemia and IR and accelerates the progression of atherosclerosis [20]. A previous study based on data from the National Metabolic Management Center also reported a positive association between TyG‐WHR and LEAD in adults with T2DM [21], but did not examine the dose–response relationship.

TyG‐WC, TyG‐WHtR and TyG‐NC exhibited J‐shaped associations with LEAD. For TyG‐WC, the RCS inflection point was 810.30, with cut‐off values of 773.63 and 844.00; the odds of LEAD increased when TyG‐WC ≥ 844.00. A study using NHANES data similarly found a nonlinear association between TyG‐WC and heart failure risk in individuals with prediabetes and diabetes, with a cut‐off of 814 [16], which is close to our upper cut‐off of 844.00. LEAD and heart failure are both macrovascular diseases; lower extremity vascular involvement tends to occur earlier in the disease course and can serve as an indicator of coronary and cerebrovascular disease [22]. For TyG‐WHtR, the RCS inflection point was 4.55, with only an upper cut‐off of 5.10; a TyG‐WHtR > 5.10 was associated with an approximately 2.5‐fold increase in the odds of LEAD. WHtR is considered a superior central obesity indicator in Chinese populations; an analysis based on CHARLS data found that TyG‐WHtR was more strongly associated with incident cardiovascular disease than TyG‐WC in middle‐aged and older adults [23]. For TyG‐NC, the inflection point was 334.56, with cut‐off values of 302.26 and 359.09; a modest increase in odds was observed within the interval of 302.26–359.09 (OR = 1.023, 95% CI: 1.000–1.047, *p* = 0.049), with a further increase at ≥ 359.09 (OR = 1.007, 95% CI: 1.002–1.013, *p* = 0.011). NC is closely correlated with WC and has been proposed as a simple screening indicator for central obesity [24]; NC is also positively associated with carotid intima‐media thickness and is comparable to WC in identifying subclinical atherosclerosis [25], supporting the plausibility of the similar nonlinear patterns observed for TyG‐NC and TyG‐WC.

In sex‐stratified analyses, only TyG‐WHR maintained a nonlinear association with LEAD in males, with an approximately 1.9‐fold increase in odds when TyG‐WHR ≥ 8.90; all other indices showed linear positive associations, among which TyG‐BMI and TyG‐WHtR exhibited the most robust associations. No significant association was observed for the TyG index in males. In females, multiple indices displayed nonlinear associations; however, with only 48 LEAD events in this subgroup, the RCS curves had wide confidence intervals at the tails, and the stability of model fitting was limited. These findings should therefore be regarded as exploratory. To formally assess whether these sex differences were statistically significant, we performed interaction analyses by including product terms between each index and sex in the total population models. The results showed significant effect modification by sex for the TyG index (*p* for interaction = 0.019) and TyG‐WHR (*p* for interaction = 0.024), whereas the interaction term for TyG‐BMI and sex was not significant (p for interaction = 0.159), indicating that the strength of the association between TyG‐BMI and LEAD was relatively consistent between males and females.

ROC analysis showed that, in the unadjusted model, TyG‐BMI had the highest discriminatory ability (AUC = 0.623), followed by TyG‐WHtR (0.591), TyG‐NC (0.584), TyG‐WC (0.579), TyG‐WHR (0.546) and the TyG index (0.507). After Bonferroni correction, it was demonstrated that the AUC of TyG‐BMI was significantly higher than those of the TyG index, TyG‐WC and TyG‐WHR (all Bonferroni *p* < 0.05). In sex‐stratified analyses, the AUC of TyG‐BMI in males was 0.660, significantly outperforming all other indices (all Bonferroni *p* < 0.05); in females, only the AUC of the TyG index reached statistical significance (*p* = 0.027). After adjustment for all covariates, the AUC of TyG‐BMI was 0.699 in the total population and 0.706 in males. In females, the highest adjusted AUC was observed for TyG‐WHtR (0.772), followed by TyG‐BMI (0.768), although no significant differences in adjusted AUCs were found in any pairwise comparisons in either the total population or females.

The superior discriminatory ability of TyG‐BMI may be attributable to the fact that BMI better reflects overall body volume and weight, which are closely related to blood viscosity and blood volume, and consequently to blood pressure [26]. In addition, BMI may correlate more effectively with brachial‐ankle pulse wave velocity [27]; both ABI and baPWV are reliable indicators of arterial stiffness and the extent of atherosclerosis [28]. In the present study, the AUC of TyG‐BMI was 0.623, which represents a moderate level of discrimination. After incorporating traditional cardiovascular risk factors, the differences in AUCs among the adjusted models narrowed, suggesting that the incremental discriminatory ability of any single index is limited. Therefore, TyG‐BMI should be regarded as a convenient discriminative indicator calculable from routine clinical data; its value lies in rapidly stratifying T2DM patients based on their current probability of having LEAD, thereby identifying those who require immediate ABI testing.

With respect to the mechanism underlying the superiority of TyG‐BMI over the TyG index alone, we consider that it primarily reflects the biological synergy between IR and obesity. In the obese state, adipose tissue oversecretes pro‐inflammatory cytokines such as TNF‐α and IL‐6, which directly induce IR through activation of the NF‐κB and JNK signalling pathways [29]; IR, in turn, impairs the anti‐lipolytic effect of insulin, leading to a massive release of free fatty acids and their ectopic deposition, further exacerbating lipid metabolism disorders and systemic inflammation [30]. These two processes form a vicious cycle that jointly accelerates the progression of atherosclerosis. The TyG index primarily reflects hepatic IR, whereas BMI reflects the overall degree of adiposity; their combination simultaneously captures both the severity of IR and the burden of obesity, thereby providing more comprehensive risk information than either index alone. In a subgroup analysis of a national cohort study, Tang et al. [31] found that in patients with T2DM, only TyG‐BMI was significantly associated with incident heart disease (highest quartile HR = 1.86, 95% CI: 1.02–3.40), whereas the TyG index alone showed no statistically significant association (HR = 0.96, 95% CI: 0.54–1.69), providing direct evidence supporting the advantage of the combined index in individuals with diabetes. A meta‐analysis also confirmed that an elevated TyG index is significantly associated with an increased risk of atherosclerotic cardiovascular disease [32], indirectly supporting the rationale for combined index assessment strategies.

Notably, although the strength of the association between TyG‐BMI and LEAD did not differ significantly by sex (*p* for interaction = 0.159), its discriminatory ability showed a sex difference (AUC 0.660 in males, 0.539 in females). This sex difference warrants further investigation. It may be attributable to several sex‐specific mechanisms. Sex differences in fat distribution constitute an important anatomical basis. At equivalent BMI levels, males tend to have a greater visceral adipose tissue area, whereas females tend to store fat subcutaneously in the gluteofemoral region [33]. Visceral adipose tissue exhibits higher lipolytic activity than subcutaneous adipose tissue and releases free fatty acids more readily into the portal circulation, directly exacerbating hepatic IR; subcutaneous adipose tissue, by contrast, serves a metabolic buffering function, more effectively taking up and storing circulating free fatty acids and thereby mitigating ectopic fat deposition [33, 34]. Consequently, TyG‐BMI, as the product of the TyG index and BMI, may more accurately capture the synergistic metabolic damage of IR and obesity in males, who are characterized by predominant visceral fat accumulation, thereby yielding superior discriminatory ability for LEAD. In addition, sex hormone‐mediated differential vascular protection may also play a role. Premenopausal females are protected by oestrogen, which activates endothelial nitric oxide synthase to promote nitric oxide production, maintains vasodilation and inhibits the expression of inflammatory cytokines and vascular smooth muscle cell proliferation [35]. This protective effect may partially offset the deleterious impact of metabolic abnormalities on the lower extremity vasculature, such that the degree of vascular damage is less pronounced in females at equivalent levels of metabolic disturbance, thereby attenuating the ability of TyG‐BMI to distinguish affected from unaffected individuals. Furthermore, with only 48 LEAD events in the female subgroup, limited statistical power may also have contributed to the instability or underestimation of the AUC.

The TyG index and obesity indices involved in this study are all readily obtainable from routine physical examinations, ensuring their convenient clinical application. In addition, the use of RCS models allowed for a more precise characterization of the continuous associations between these indices and LEAD, thereby avoiding the information loss that typically results from categorizing continuous variables. This study has several limitations. First, the cross‐sectional design can only evaluate associations and discriminatory ability for identifying LEAD, not causality. Second, the female subgroup had only 48 LEAD events, limiting statistical power and leading to wide confidence intervals and unstable AUC estimates; these findings are exploratory and require validation in larger female cohorts. Third, the 99.4% rate of glucose‐lowering medication use provided no between‐group variation for adjustment, and data on drug types/doses were not collected, precluding analysis of their potential impact. Fourth, participants were recruited from two tertiary hospitals in a single region, which may limit generalizability.

## 5 Conclusion

In conclusion, this study found that TyG‐BMI, TyG‐WHtR and elevated levels of the TyG index (≥ 9.52), TyG‐WC (≥ 844.00), TyG‐WHR (≥ 8.80) and TyG‐NC (≥ 359.09) were positively associated with LEAD in patients with T2DM. Among the evaluated TyG‐derived indices, TyG‐BMI exhibited the relatively highest discriminatory ability for LEAD; however, the absolute discrimination remained modest (AUC: 0.623). TyG‐BMI may serve as a convenient tool for stratifying LEAD using routine clinical data, particularly for identifying male T2DM patients with a high likelihood of having the condition. The strength of the association between certain indices and LEAD differed by sex, which should be taken into account in clinical interpretation. Future multicenter, large‐sample prospective cohort studies are warranted to validate the predictive value of these indices for LEAD in patients with T2DM.

## Author Contributions

**Rong Zhang:** formal analysis, writing – original draft, methodology. **Guilin Liu:** formal analysis, software, data curation. **Qian Cui:** validation, investigation. **Dan Fang:** software, data curation, formal analysis. **Zhenzhen Sun:** investigation, validation. **Xiaodong Chen:** resources. **Rendong Zheng:** resources. **Zhenxiu Gao:** conceptualization, project administration, supervision, writing – review and editing. **Xiaodan Yuan:** conceptualization, supervision, project administration, writing – review and editing, funding acquisition.

## Funding

This work was supported by Jiangsu Province Academy of Traditional Chinese Medicine Ym2023048.

## Conflicts of Interest

The authors declare no conflicts of interest.

## Supporting information


**Figure S1:** Restricted cubic spline curves for association of TyG index and its combinations with obesity indices with LEAD (sex‐stratified).
**Figure S2:** Receiver operating characteristic curves showing discriminatory ability of TyG index and its combinations with obesity indices for LEAD (A: total population; B: male; C: female).

**Table S1:** Sex‐stratified association of TyG index and its combinations with obesity indices with LEAD in patients with T2DM.
**Table S2:** Pairwise comparisons of AUC for discriminatory ability of TyG index and its combinations with obesity indices for LEAD (unadjusted).
**Table S3:** Pairwise comparisons of AUC for discriminatory ability of TyG index and its combinations with obesity indices for LEAD (adjusted for covariates).

## Data Availability

The data that support the findings of this study are available on request from the corresponding author. The data are not publicly available due to privacy or ethical restrictions.

## References

[1] B. Zhang , H. Zhang , H. Gong , et al., “Trends and Potential Risk Factors of Lower Extremity Peripheral Arterial Disease: Results From the Global Burden of Disease Study 2021,” Pulse 13, no. 1 (2025): 140–156. 41246568 10.1159/000547795 PMC12618024

[2] P. Song , D. Rudan , Y. Zhu , et al., “Global, Regional, and National Prevalence and Risk Factors for Peripheral Artery Disease in 2015: An Updated Systematic Review and Analysis,” Lancet Global Health 7, no. 8 (2019): e1020–e1030. 31303293 10.1016/S2214-109X(19)30255-4

[3] X. M. Zhang , X. W. Ran , Z. R. Xu , et al., “Epidemiological Characteristics of Lower Extremity Arterial Disease in Chinese Diabetes Patients at High Risk: A Prospective, Multicenter, Cross‐Sectional Study,” Journal of Diabetes and Its Complications 32, no. 2 (2018): 150–156. 29191431 10.1016/j.jdiacomp.2017.10.003

[4] Chinese Diabetes Society , “Guideline for the Prevention and Treatment of Diabetes Mellitus in China (2024 Edition),” Chinese Journal of Diabetes Mellitus 17, no. 1 (2025): 16–139.

[5] A. T. Hirsch , T. P. Murphy , M. B. Lovell , et al., “Gaps in Public Knowledge of Peripheral Arterial Disease ‐ The First National PAD Public Awareness Survey,” Circulation 116, no. 18 (2007): 2086–2094. 17875966 10.1161/CIRCULATIONAHA.107.725101

[6] A. W. Aday and K. Matsushita , “Epidemiology of Peripheral Artery Disease and Polyvascular Disease,” Circulation Research 128, no. 12 (2021): 1818–1832. 34110907 10.1161/CIRCRESAHA.121.318535 PMC8202714

[7] A. Raghav , Z. A. Khan , R. K. Labala , et al., “Financial Burden of Diabetic Foot Ulcers to World: A Progressive Topic to Discuss Always,” Therapeutic Advances in Endocrinology and Metabolism 9, no. 1 (2018): 29–31. 29344337 10.1177/2042018817744513 PMC5761954

[8] A. H. Wang , Z. R. Xu , and L. N. Ji , “Clinical Characteristics and Medical Costs of Diabetics With Amputation at Central Urban Hospitals in China,” National Medical Journal of China 92, no. 4 (2012): 224–227. 22490790

[9] M. D. Gerhard‐Herman , H. L. Gornik , C. Barrett , et al., “2016 AHA/ACC Guideline on the Management of Patients With Lower Extremity Peripheral Artery Disease: Executive Summary a Report of the American College of Cardiology/American Heart Association Task Force on Clinical Practice Guidelines,” Journal of the American College of Cardiology 69, no. 11 (2017): 1465. 27851991 10.1016/j.jacc.2016.11.008

[10] V. K. Ramdas Nayak , P. Satheesh , M. T. Shenoy , et al., “Triglyceride Glucose (TyG) Index: A Surrogate Biomarker of Insulin Resistance,” Journal of the Pakistan Medical Association 72, no. 5 (2022): 986–988. 35713073 10.47391/JPMA.22-63

[11] I. Desormais , V. Aboyans , M. Guerchet , et al., “Body Mass Index and Peripheral Arterial Disease, a “U‐Shaped” Relationship in Elderly African Population—The EPIDEMCA Study,” VASA 49, no. 1 (2020): 50–56. 31621522 10.1024/0301-1526/a000825

[12] Y. Liu , L. Chang , M. Wu , et al., “Triglyceride Glucose Index Was Associated With the Risk of Peripheral Artery Disease,” Angiology 73, no. 7 (2022): 655–659. 35077252 10.1177/00033197211070644

[13] Y. H. Wei , C. Y. Liu , Y. Y. Liu , et al., “The Association Between Time in the Glucose Target Range and Abnormal Ankle‐Brachial Index: A Cross‐Sectional Analysis,” Cardiovascular Diabetology 21, no. 1 (2022): 281. 36514151 10.1186/s12933-022-01718-y PMC9746002

[14] M. Y. Fan , J. Lyu , and P. P. He , “Chinese Guidelines for Data Processing and Analysis Concerning the International Physical Activity Questionnaire,” Chinese Journal of Epidemiology 35, no. 8 (2014): 961–964. 25376692

[15] A. S. Levey , L. A. Stevens , C. H. Schmid , et al., “A New Equation to Estimate Glomerular Filtration Rate,” Annals of Internal Medicine 150, no. 9 (2009): 604–612. 19414839 10.7326/0003-4819-150-9-200905050-00006 PMC2763564

[16] D. Z. Zheng , J. M. Cai , S. F. Xu , et al., “The Association of Triglyceride‐Glucose Index and Combined Obesity Indicators With Chest Pain and Risk of Cardiovascular Disease in American Population With Pre‐Diabetes or Diabetes,” Frontiers in Endocrinology 15 (2024): 1471535. 39309107 10.3389/fendo.2024.1471535 PMC11412814

[17] S. Zhao , S. K. Yu , C. Chi , et al., “Association Between Macro‐ and Microvascular Damage and the Triglyceride Glucose Index in Community‐Dwelling Elderly Individuals: The Northern Shanghai Study,” Cardiovascular Diabetology 18, no. 1 (2019): 95. 31345238 10.1186/s12933-019-0898-x PMC6657056

[18] P. Ning , J. Zeng , Q. Feng , et al., “Triglyceride‐Glucose Index as a Predictor of Lower Extremity Arterial Disease in Patients With Diabetes: A Hospitalized Population Retrospective Study,” Annals of Vascular Surgery 98 (2024): 173–181. 37802143 10.1016/j.avsg.2023.08.013

[19] N. Stefan , F. Schick , and H. Häring , “Causes, Characteristics, and Consequences of Metabolically Unhealthy Normal Weight in Humans,” Cell Metabolism 26, no. 2 (2017): 292–300. 28768170 10.1016/j.cmet.2017.07.008

[20] I. J. Neeland , R. Ross , J. P. Després , et al., “Visceral and Ectopic Fat, Atherosclerosis, and Cardiometabolic Disease: A Position Statement,” Lancet Diabetes & Endocrinology 7, no. 9 (2019): 715–725. 31301983 10.1016/S2213-8587(19)30084-1

[21] Y. Miao , Y. Wang , Y. Wang , et al., “The Association Between Triglyceride‐Glucose Index and Its Combination With Obesity Indicators and Lower Extremity Peripheral Artery Disease in Patients With Type 2 Diabetes Mellitus: A Cross‐Sectional Study,” Diabetes, Metabolic Syndrome and Obesity: Targets and Therapy 17 (2024): 2607–2617. 38953012 10.2147/DMSO.S469692 PMC11216433

[22] X. H. Pang , J. Han , W. L. Ye , et al., “Lower Extremity Peripheral Arterial Disease Is an Independent Predictor of Coronary Heart Disease and Stroke Risks in Patients With Type 2 Diabetes Mellitus in China,” International Journal of Endocrinology 2017 (2017): 9620513. 28607554 10.1155/2017/9620513 PMC5457753

[23] C. C. Cui , Y. T. Qi , J. Y. Song , et al., “Comparison of Triglyceride Glucose Index and Modified Triglyceride Glucose Indices in Prediction of Cardiovascular Diseases in Middle Aged and Older Chinese Adults,” Cardiovascular Diabetology 23, no. 1 (2024): 185. 38812015 10.1186/s12933-024-02278-z PMC11138075

[24] T. Anothaisintawee , N. Sansanayudh , S. Thamakaison , et al., “Neck Circumference as an Anthropometric Indicator of Central Obesity in Patients With Prediabetes: A Cross‐Sectional Study,” BioMed Research International 2019 (2019): 4808541. 31281839 10.1155/2019/4808541 PMC6590547

[25] Y. T. Xu , C. H. Jian , X. J. Ma , et al., “Comparison of Neck and Waist Circumferences for Identifying Subclinical Atherosclerosis in a Community‐Based Population,” Diabetes/Metabolism Research and Reviews 37, no. 5 (2021): e3382. 32628319 10.1002/dmrr.3382

[26] X. H. Hou , J. M. Lu , J. P. Weng , et al., “Impact of Waist Circumference and Body Mass Index on Risk of Cardiometabolic Disorder and Cardiovascular Disease in Chinese Adults: A National Diabetes and Metabolic Disorders Survey,” PLoS One 8, no. 3 (2013): e57319. 23520466 10.1371/journal.pone.0057319 PMC3592870

[27] S. M. Y. Zhulepiya , G. M. Hu , X. Chen , et al., “The Correlation of Body Mass Index and Waist Circumference in Healthy and Hypertensive People With Arterial Stiffness,” Chinese Journal of Hypertension 21, no. 5 (2013): 477–479.

[28] R. J. Liang and R. K. Liang , “The Correlation of Blood Pressure Level With Brachial‐Ankle Pulse Wave Velocity and Ankle Brachial Index in Elderly Hypertensive Patients With Different Body Mass Index,” Chinese Journal of Hypertension 27, no. 6 (2019): 530–535.

[29] M. A. McArdle , O. M. Finucane , R. M. Connaughton , et al., “Mechanisms of Obesity‐Induced Inflammation and Insulin Resistance: Insights Into the Emerging Role of Nutritional Strategies,” Frontiers in Endocrinology 4 (2013): 52. 23675368 10.3389/fendo.2013.00052 PMC3650620

[30] V. T. Samuel and G. I. Shulman , “The Pathogenesis of Insulin Resistance: Integrating Signaling Pathways and Substrate Flux,” Journal of Clinical Investigation 126, no. 1 (2016): 12–22. 26727229 10.1172/JCI77812 PMC4701542

[31] X. D. Tang , K. X. Zhang , and R. He , “The Association of Triglyceride‐Glucose and Triglyceride‐Glucose Related Indices With the Risk of Heart Disease in a National Cohort Study,” Cardiovascular Diabetology 24, no. 1 (2025): 54. 39915784 10.1186/s12933-025-02621-y PMC11803996

[32] X. B. Ding , X. Z. Wang , J. Wu , et al., “Triglyceride‐Glucose Index and the Incidence of Atherosclerotic Cardiovascular Diseases: A Meta‐Analysis of Cohort Studies,” Cardiovascular Diabetology 20, no. 1 (2021): 76. 33812373 10.1186/s12933-021-01268-9 PMC8019501

[33] K. Karastergiou , S. R. Smith , A. S. Greenberg , et al., “Sex Differences in Human Adipose Tissues – The Biology of Pear Shape,” Biology of Sex Differences 3, no. 1 (2012): 13. 22651247 10.1186/2042-6410-3-13 PMC3411490

[34] G. H. Goossens , J. W. E. Jocken , and E. E. Blaak , “Sexual Dimorphism in Cardiometabolic Health: The Role of Adipose Tissue, Muscle and Liver,” Nature Reviews Endocrinology 17, no. 1 (2021): 47–66. 10.1038/s41574-020-00431-8 33173188

[35] M. E. Mendelsohn and R. H. Karas , “The Protective Effects of Estrogen on the Cardiovascular System,” New England Journal of Medicine 340, no. 23 (1999): 1801–1811. 10362825 10.1056/NEJM199906103402306

