# Supplementary material for: The Triglyceride‐Glucose Index Combined With Obesity Indices and Lower Extremity Artery Disease in Type 2 Diabetes: A Sex‐Stratified Analysis
Source: Endocrinol Diabetes Metab. 2026 Jul 7;9(4):e70278. doi: 10.1002/edm2.70278 (PMC13341960; doi:10.1002/edm2.70278)
Supplement: Supplementary file 1 — Figure S1: Restricted cubic spline curves for association of TyG index and its combinations with obesity indices with LEAD (sex‐stratified). Figure S2: Receiver operating characteristic curves showing discriminatory ability of TyG index and its combinations with obesity indices for LEAD (A: total population; B: male; C: female). [file EDM2-9-e70278-s002.docx]

**Supplementary Figure 1** | Restricted cubic spline curves for association of TyG index and its combinations with obesity indices with LEAD (sex-stratified)


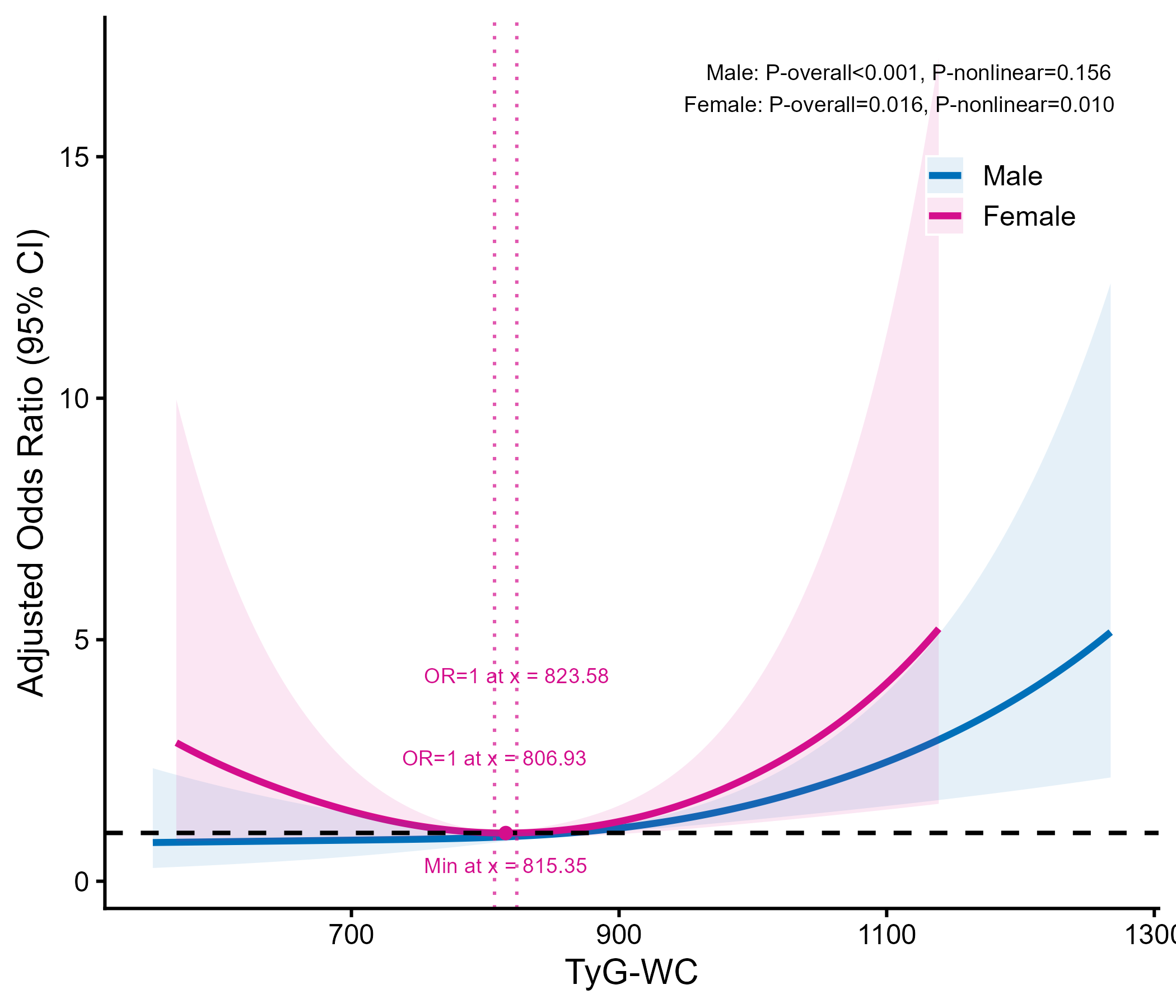


C


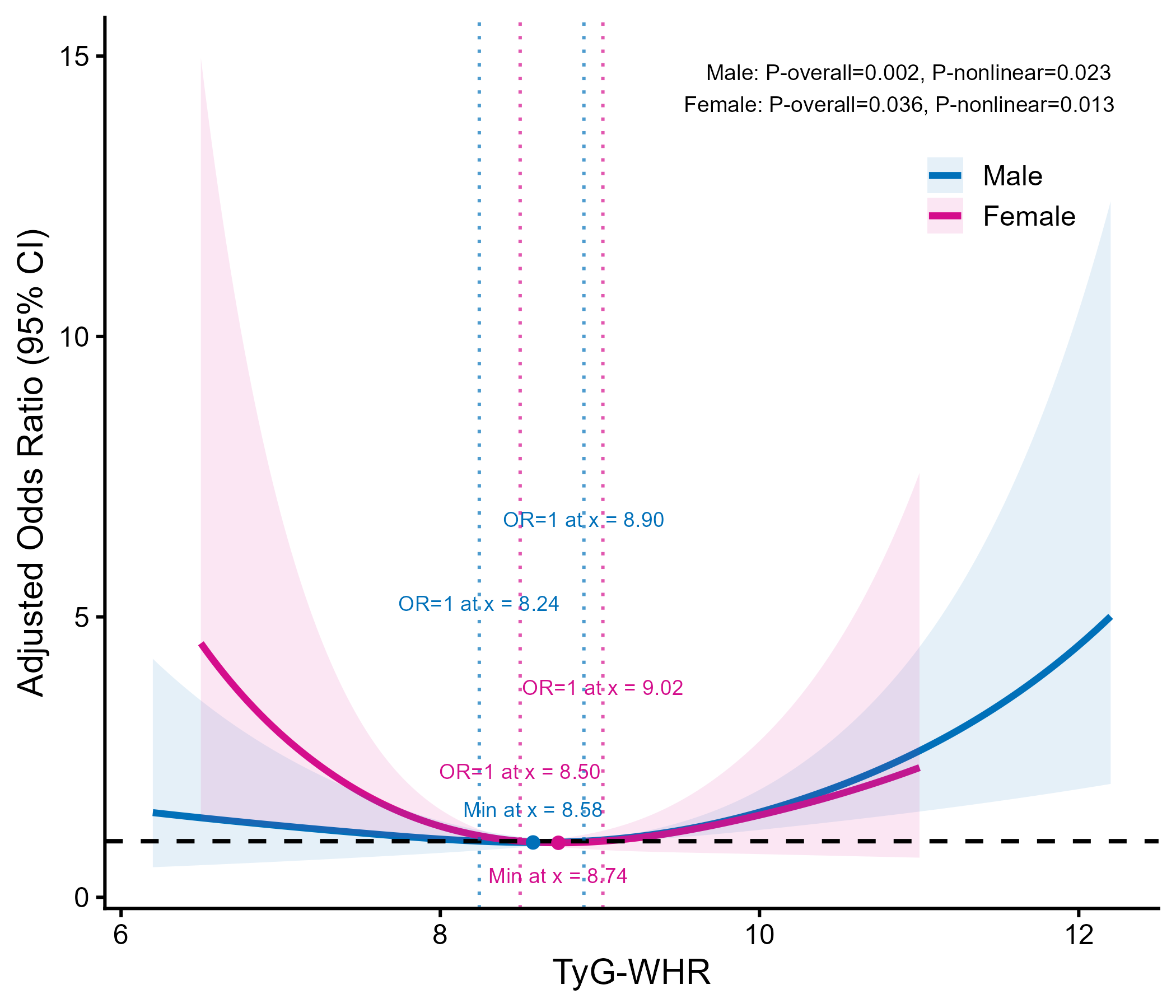


D


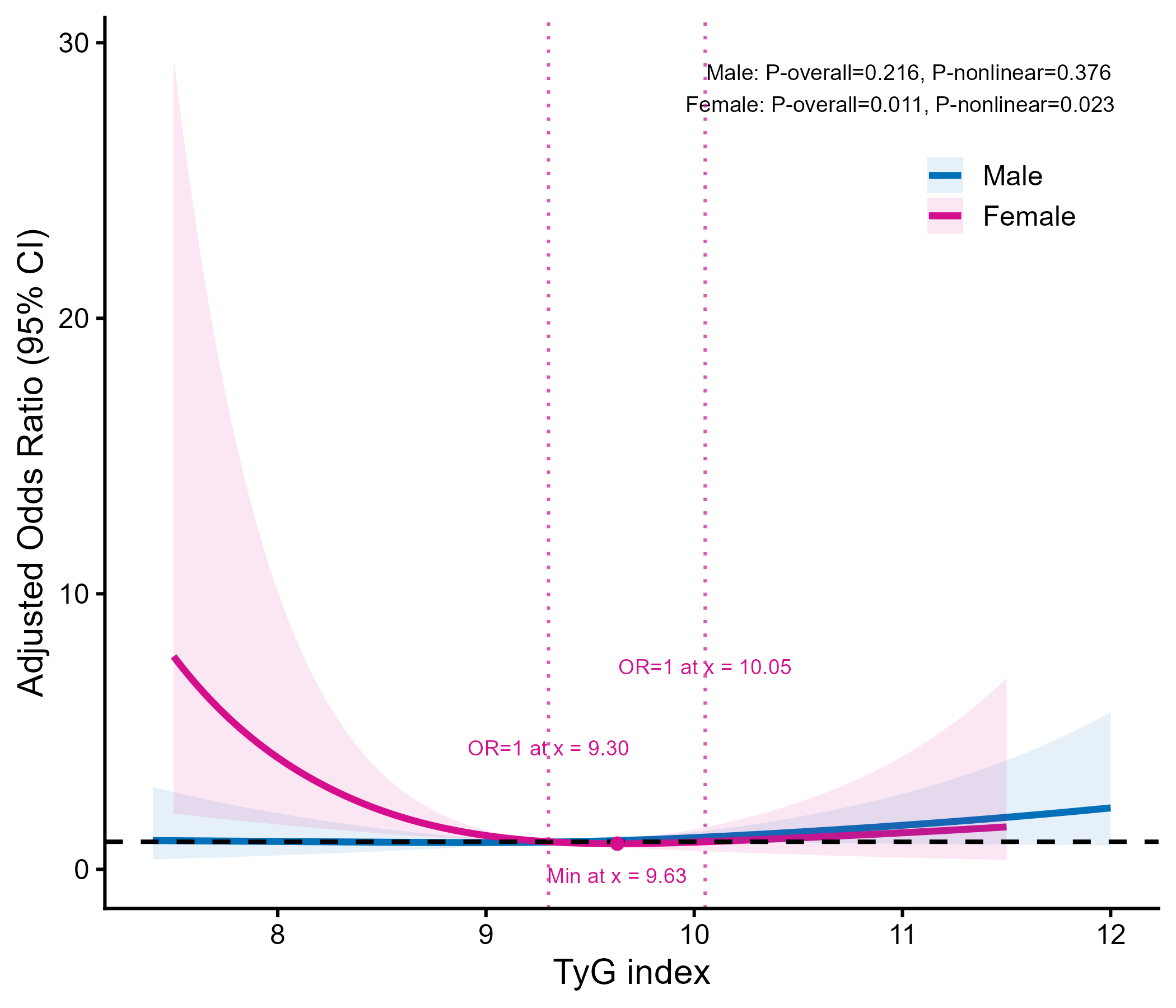


A


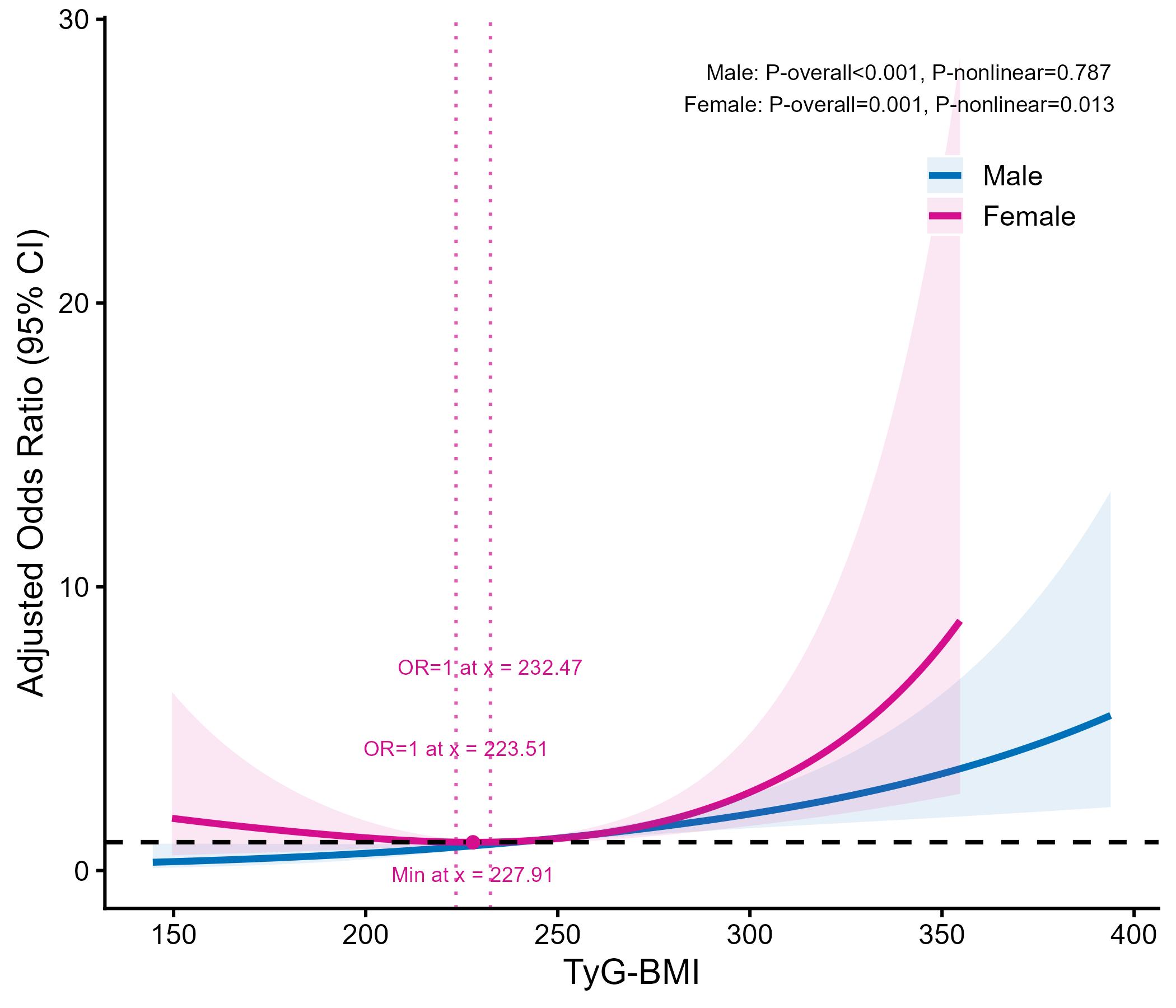


B


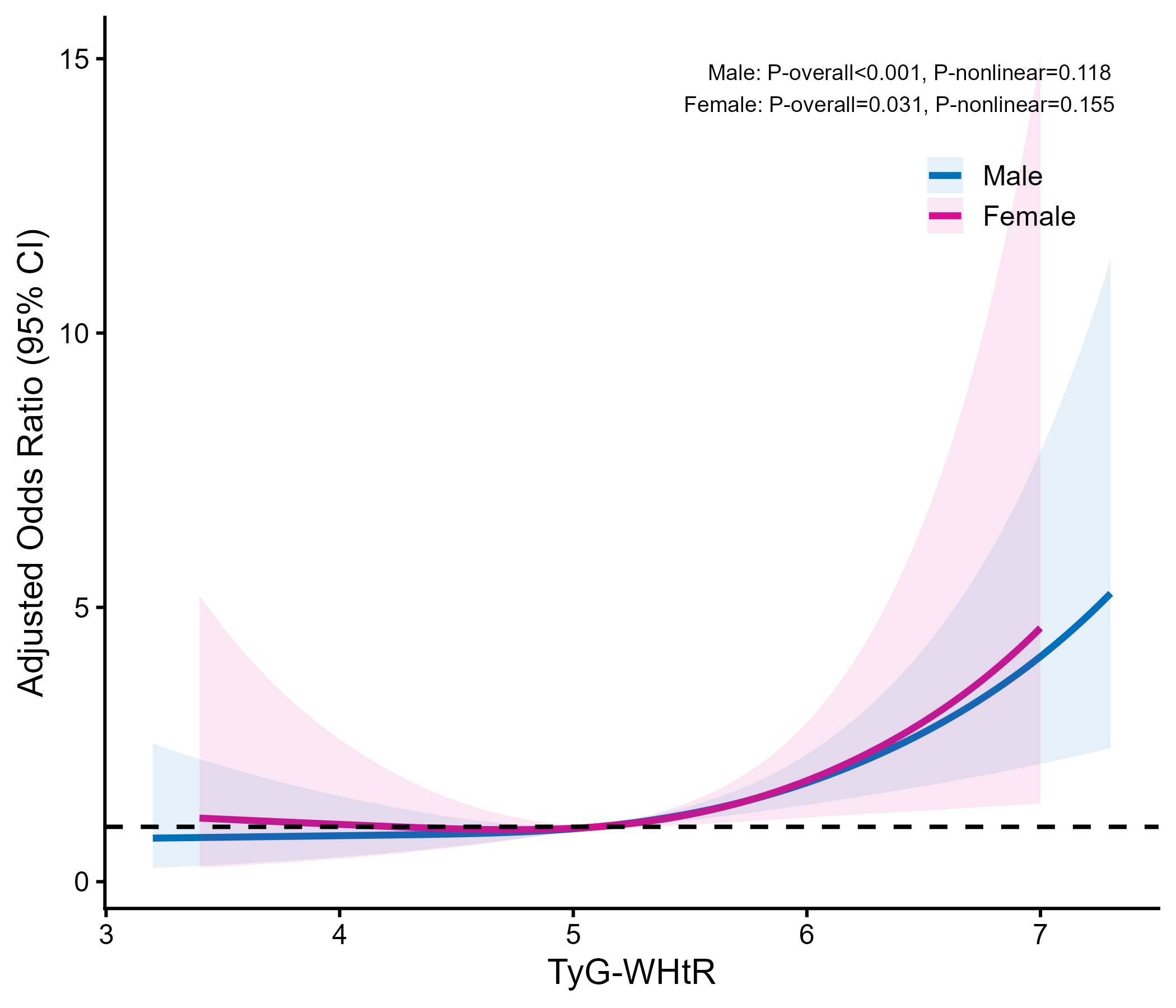


E


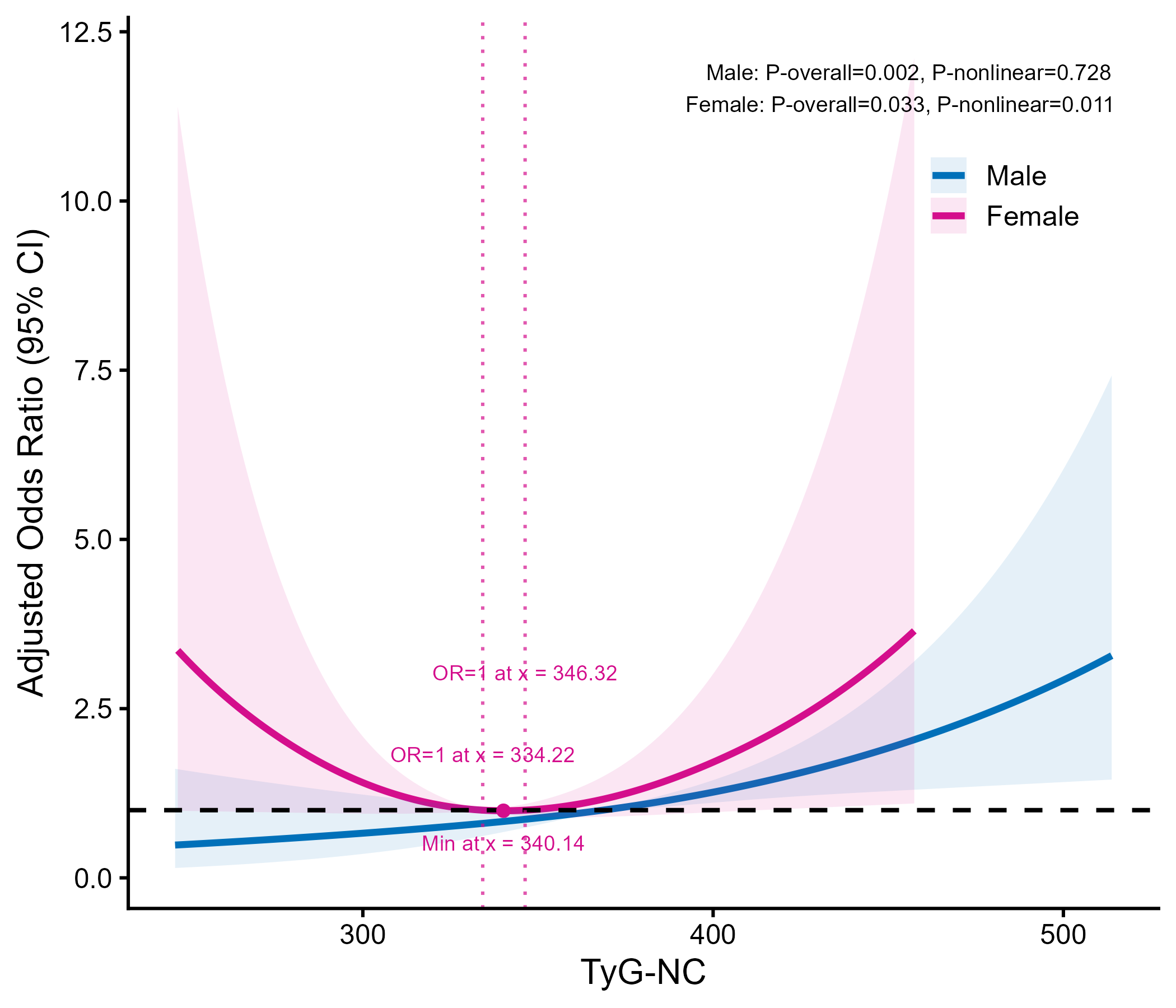


F

*Note*: Adjusted for age, educational attainment, duration of diabetes, smoking status, alcohol consumption, hypertension, hyperlipidemia, lipid-lowering medication, HbA1c, HDL-c, LDL-c, DBP, SBP, UACR, eGFR, physical activity and sedentary time. Sex-stratified models were not adjusted for sex.

**Supplementary Figure 2** | Receiver operating characteristic curves showing discriminatory ability of TyG index and its combinations with obesity indices for LEAD (A: total population; B: male; C: female)


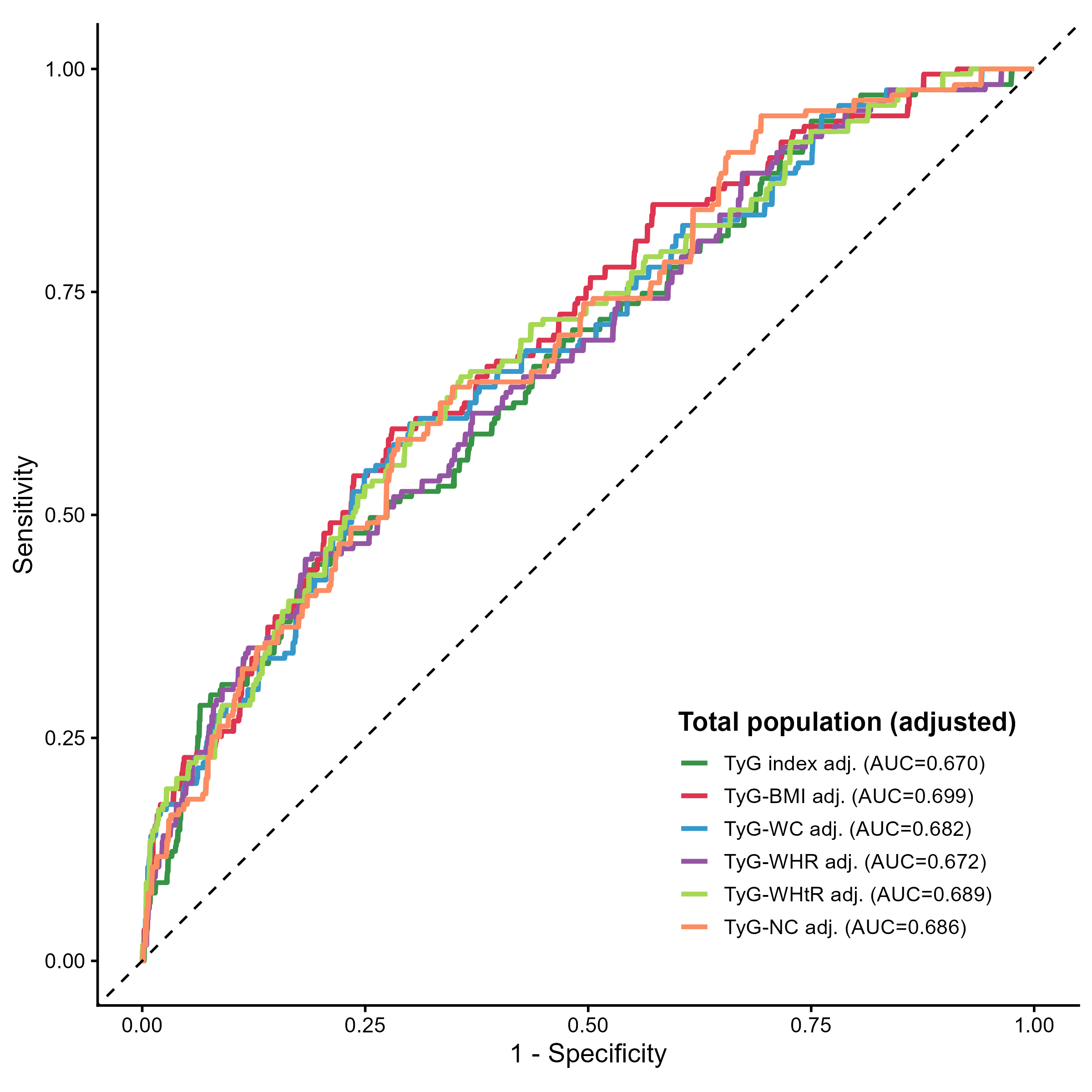


A


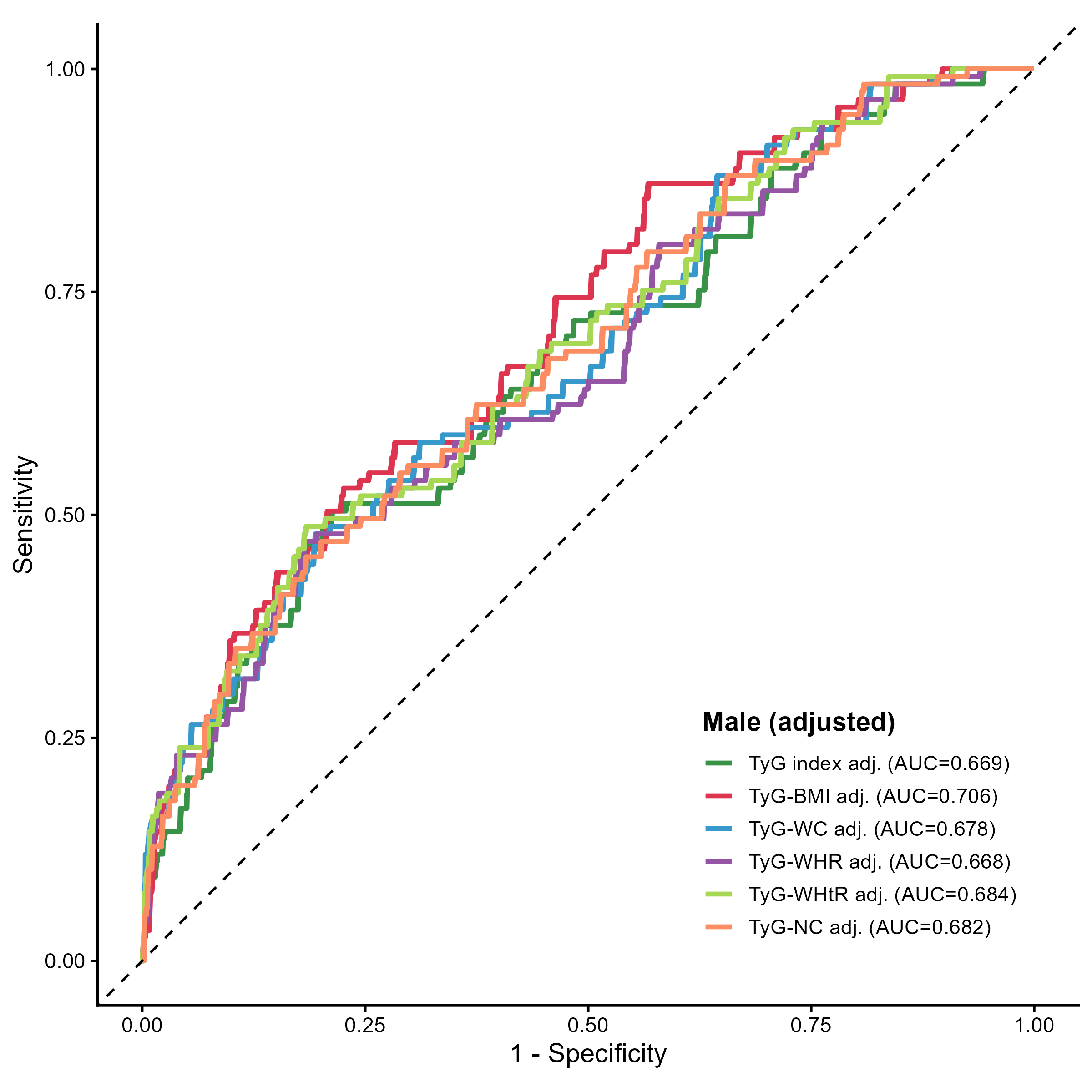


B


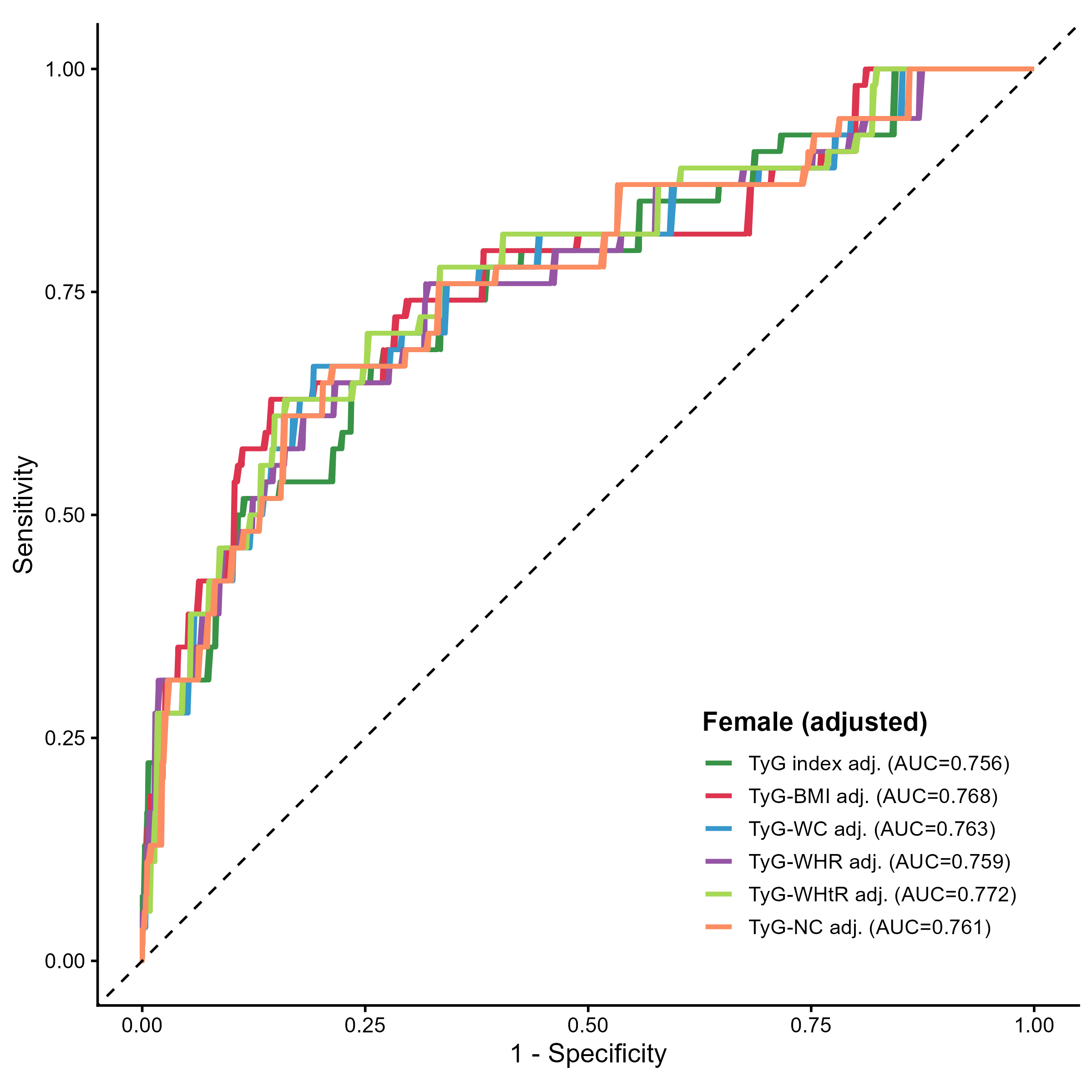


C

*Note*: Adjusted for sex, age, educational attainment, duration of diabetes, smoking status, alcohol consumption, hypertension, hyperlipidemia, lipid-lowering medication, HbA1c, HDL-c, LDL-c, DBP, SBP, UACR, eGFR, physical activity and sedentary time. Sex-stratified models were not adjusted for sex.
