# Supplementary material for: The Triglyceride‐Glucose Index Combined With Obesity Indices and Lower Extremity Artery Disease in Type 2 Diabetes: A Sex‐Stratified Analysis
Source: Endocrinol Diabetes Metab. 2026 Jul 7;9(4):e70278. doi: 10.1002/edm2.70278 (PMC13341960; doi:10.1002/edm2.70278)
Supplement: Supplementary file 2 — Table S1: Sex‐stratified association of TyG index and its combinations with obesity indices with LEAD in patients with T2DM. Table S2: Pairwise comparisons of AUC for discriminatory ability of TyG index and its combinations with obesity indices for LEAD (unadjusted). Table S3: Pairwise comparisons of AUC for discriminatory ability of TyG index and its combinations with obesity indices for LEAD (adjusted for covariates). [file EDM2-9-e70278-s001.docx]

**Supplementary Table 1** | Sex-stratified association of TyG index and its combinations with obesity indices with LEAD in patients with T2DM

| Variable | N (Events) | Unadjusted | | Model 1^*^ | |
| --- | --- | --- | --- | --- | --- |
|  |  | OR（95%CI） | *p* | OR（95%CI） | *p* |
| Male |  |  |  |  |  |
| TyG | 1593 (123) | 1.248 (1.003, 1.547) | 0.045 | 1.207 (0.935, 1.551) | 0.145 |
| TyG-BMI | 1593 (123) | 1.010 (1.007, 1.014) | <0.001 | 1.012 (1.007, 1.016) | <0.001 |
| TyG-WC | 1593 (123) | 1.003 (1.001, 1.004) | <0.001 | 1.003 (1.001, 1.005) | <0.001 |
| TyG-WHR ≤8.24 | 405 (27) | 1.180 (0.592, 2.629) | 0.661 | 0.950 (0.398, 2.449) | 0.911 |
| TyG-WHR ≥8.90 | 822 (75) | 1.630 (1.199, 2.188) | 0.001 | 1.912 (1.308, 2.793) | <0.001 |
| TyG-WHR 8.24-8.90 | 366 (21) | 1.350 (0.147, 12.910) | 0.791 | 4.325 (0.284, 83.343) | 0.305 |
| TyG-WHtR | 1593 (123) | 1.682 (1.338, 2.112) | <0.001 | 1.765 (1.353, 2.307) | <0.001 |
| TyG-NC | 1593 (123) | 1.007 (1.004, 1.011) | <0.001 | 1.008 (1.003, 1.012) | <0.001 |
| Female |  |  |  |  |  |
| TyG ≤9.30 | 429 (33) | 0.302 (0.155, 0.589) | <0.001 | 0.680 (0.235, 1.952) | 0.471 |
| TyG ≥10.05 | 105(9) | 1.088 (0.210, 3.896) | 0.907 | — | — |
| TyG 9.30-10.05 | 297 (6) | 0.389 (0.012, 9.603) | 0.573 | — | — |
| TyG-BMI ≤223.51 | 339 (21) | 0.989 (0.971, 1.009) | 0.249 | 0.997 (0.967, 1.031) | 0.855 |
| TyG-BMI ≥232.47 | 399 (21) | 1.016 (1.006, 1.025) | 0.002 | 1.022 (1.009, 1.036) | 0.001 |
| TyG-BMI 223.51-232.47 | 93 (6) | 0.853 (0.563, 1.217) | 0.400 | — | — |
| TyG-WC ≤806.93 | 453 (30) | 0.995 (0.990, 1.001) | 0.099 | 1.001 (0.993, 1.010) | 0.795 |
| TyG-WC ≥823.58 | 348 (18) | 1.005 (1.000, 1.009) | 0.026 | 1.004 (0.998, 1.009) | 0.147 |
| TyG-WC 806.93-823.58 | 30 (0) | — | — | — | — |
| TyG-WHR ≤8.50 | 450 (30) | 0.432 (0.244, 0.781) | 0.004 | 0.701 (0.213, 2.313) | 0.554 |
| TyG-WHR ≥9.02 | 234 (12) | 1.272 (0.546, 2.634) | 0.542 | 0.381 (0.055, 1.689) | 0.260 |
| TyG-WHR 8.50-9.02 | 147 (6) | — | — | — | — |
| TyG-WHtR | 831 (48) | 1.233 (0.860, 1.757) | 0.250 | 1.616 (1.032, 2.520) | 0.034 |
| TyG-NC ≤334.22 | 468 (30) | 0.985 (0.971, 1.000) | 0.039 | 0.998 (0.959, 1.035) | 0.905 |
| TyG-NC ≥346.32 | 279 (15) | 1.014 (1.001, 1.025) | 0.025 | 1.009 (0.992, 1.024) | 0.286 |
| TyG-NC 334.22-346.32 | 84 (3) | 0.733 (0.417, 1.059) | 0.165 | — | — |

*Note*: Model 1^*^ was adjusted for age, educational attainment, duration of diabetes, hypertension, hyperlipidemia, lipid-lowering medication, smoking status, alcohol consumption, HbA1c, HDL-c, LDL-c, DBP, SBP, UACR, eGFR, physical activity, and sedentary behavior (sex was not adjusted for as the analysis was stratified by sex). "—" indicates that data are not presented because the model failed to provide stable estimates due to insufficient sample size or too few outcome events in that stratum.

**Supplementary Table 2** | Pairwise comparisons of AUC for discriminatory ability of TyG index and its combinations with obesity indices for LEAD (unadjusted)

| Comparison | Total (N=2424) | | | Male (N=1593) | | | Female (N=831) | | |
| --- | --- | --- | --- | --- | --- | --- | --- | --- | --- |
|  | Delta_AUC | Raw_*p* | Bonferroni_*p* | Delta_AUC | Raw_*p* | Bonferroni_*p* | Delta_AUC | Raw_*p* | Bonferroni_*p* |
| TyG index vs TyG-BMI | -0.116 | <0.0001 | <0.0001 | -0.113 | <0.0001 | <0.0001 | 0.051 | 0.5377 | 1.0000 |
| TyG index vs TyG-WC | -0.072 | <0.0001 | <0.0001 | -0.061 | 0.0008 | 0.0120 | 0.097 | 0.0023 | 0.0340 |
| TyG index vs TyG-WHR | -0.039 | 0.0041 | 0.0609 | -0.027 | 0.1106 | 1.0000 | 0.064 | 0.0082 | 0.1224 |
| TyG index vs TyG-WHtR | -0.084 | <0.0001 | <0.0001 | -0.072 | 0.0008 | 0.0114 | 0.061 | 0.4760 | 1.0000 |
| TyG index vs TyG-NC | -0.077 | <0.0001 | <0.0001 | -0.062 | <0.0001 | 0.0006 | 0.082 | 0.3702 | 1.0000 |
| TyG-BMI vs TyG-WC | 0.044 | <0.0001 | 0.0004 | 0.052 | <0.0001 | 0.0002 | 0.046 | 0.6178 | 1.0000 |
| TyG-BMI vs TyG-WHR | 0.077 | <0.0001 | 0.0006 | 0.086 | <0.0001 | <0.0001 | 0.012 | 0.8802 | 1.0000 |
| TyG-BMI vs TyG-WHtR | 0.032 | 0.0104 | 0.1555 | 0.041 | 0.0020 | 0.0305 | 0.010 | 0.7195 | 1.0000 |
| TyG-BMI vs TyG-NC | 0.038 | 0.0189 | 0.2831 | 0.050 | 0.0012 | 0.0179 | 0.030 | 0.4289 | 1.0000 |
| TyG-WC vs TyG-WHR | 0.033 | 0.0031 | 0.0461 | 0.034 | 0.0006 | 0.0087 | -0.033 | 0.2769 | 1.0000 |
| TyG-WC vs TyG-WHtR | -0.012 | 0.1390 | 1.0000 | -0.011 | 0.2080 | 1.0000 | -0.036 | 0.6888 | 1.0000 |
| TyG-WC vs TyG-NC | -0.005 | 0.6657 | 1.0000 | -0.001 | 0.9166 | 1.0000 | -0.015 | 0.8663 | 1.0000 |
| TyG-WHR vs TyG-WHtR | -0.045 | <0.0001 | 0.0003 | -0.045 | <0.0001 | 0.0004 | -0.003 | 0.9754 | 1.0000 |
| TyG-WHR vs TyG-NC | -0.039 | 0.0030 | 0.0447 | -0.035 | 0.0170 | 0.2547 | 0.018 | 0.8460 | 1.0000 |
| TyG-WHtR vs TyG-NC | 0.007 | 0.6760 | 1.0000 | 0.010 | 0.5857 | 1.0000 | 0.021 | 0.4565 | 1.0000 |

*Abbreviation*s: ROC, receiver operating characteristic; AUC, area under the ROC curve.

**Supplementary Table 3** | Pairwise comparisons of AUC for discriminatory ability of TyG index and its combinations with obesity indices for LEAD (adjusted for covariates)

| Comparison | Total (N=2424) | | | Male (N=1593) | | | Female (N=831) | | |
| --- | --- | --- | --- | --- | --- | --- | --- | --- | --- |
|  | Delta_AUC | Raw_*p* | Bonferroni_*p* | Delta_AUC | Raw_*p* | Bonferroni_*p* | Delta_AUC | Raw_*p* | Bonferroni_*p* |
| TyG index adj. vs TyG-BMI adj. | -0.029 | 0.0185 | 0.2770 | -0.037 | 0.0103 | 0.1541 | -0.012 | 0.3502 | 1.0000 |
| TyG index adj.vs TyG-WC adj. | -0.012 | 0.1778 | 1.0000 | -0.009 | 0.3849 | 1.0000 | -0.007 | 0.4816 | 1.0000 |
| TyG index adj.vs TyG-WHR adj. | -0.002 | 0.6502 | 1.0000 | 0.001 | 0.8870 | 1.0000 | -0.003 | 0.6153 | 1.0000 |
| TyG index adj. vs TyG-WHtR adj. | -0.019 | 0.0797 | 1.0000 | -0.015 | 0.2613 | 1.0000 | -0.016 | 0.2540 | 1.0000 |
| TyG index adj.vs TyG-NC adj. | -0.017 | 0.0549 | 0.8238 | -0.013 | 0.1229 | 1.0000 | -0.005 | 0.6112 | 1.0000 |
| TyG-BMI adj. vs TyG-WC adj. | 0.017 | 0.0047 | 0.0707 | 0.029 | 0.0002 | 0.0032 | 0.005 | 0.5105 | 1.0000 |
| TyG-BMI adj.vs TyG-WHR adj. | 0.027 | 0.0065 | 0.0973 | 0.038 | 0.0012 | 0.0173 | 0.009 | 0.4097 | 1.0000 |
| TyG-BMI adj.vs TyG-WHtR adj. | 0.010 | 0.0848 | 1.0000 | 0.023 | 0.0029 | 0.0430 | -0.003 | 0.6682 | 1.0000 |
| TyG-BMI adj. vs TyG-NC adj. | 0.012 | 0.1627 | 1.0000 | 0.025 | 0.0236 | 0.3541 | 0.008 | 0.4860 | 1.0000 |
| TyG-WC adj. vs TyG-WHR adj. | 0.010 | 0.0586 | 0.8784 | 0.010 | 0.0865 | 1.0000 | 0.004 | 0.5014 | 1.0000 |
| TyG-WC adj.vs TyG-WHtR adj. | -0.007 | 0.0520 | 0.7801 | -0.006 | 0.2542 | 1.0000 | -0.008 | 0.0719 | 1.0000 |
| TyG-WC adj. vs TyG-NC adj. | -0.005 | 0.3908 | 1.0000 | -0.004 | 0.5664 | 1.0000 | 0.003 | 0.5237 | 1.0000 |
| TyG-WHR adj.vs TyG-WHtR adj. | -0.017 | 0.0194 | 0.2916 | -0.016 | 0.0573 | 0.8599 | -0.013 | 0.2258 | 1.0000 |
| TyG-WHR adj.vs TyG-NC adj. | -0.014 | 0.0063 | 0.0944 | -0.014 | 0.0410 | 0.6150 | -0.002 | 0.7261 | 1.0000 |
| TyG-WHtR adj.vs TyG-NC adj. | 0.003 | 0.7047 | 1.0000 | 0.002 | 0.8507 | 1.0000 | 0.011 | 0.1529 | 1.0000 |

*Note*: adj. indicates adjusted model. Model was adjusted for sex, age, educational attainment, duration of diabetes, smoking status, alcohol consumption, hypertension, hyperlipidemia, lipid-lowering medication, HbA1c, HDL-c, LDL-c, DBP, SBP, UACR, eGFR, physical activity and sedentary time.

Sex-stratified models were not adjusted for sex.

*Abbreviation*s: ROC, receiver operating characteristic; AUC, area under the ROC curve.
